# Exploring a novel copper(II) semicarbazone–pyranoquinoline complex: synthesis, spectroscopic profiling, and DFT insights

**DOI:** 10.1038/s41598-025-26205-8

**Published:** 2025-11-18

**Authors:** A. A. El-Saady, Magdy A. Ibrahim, M. M. El-Nahass, Omima M. I. Adly, A. A. M. Farag, Nesma Salah

**Affiliations:** 1 Thin Film Laboratory, Physics Department, Faculty of Education, Ain Shams University, Roxy, Cairo, 11757 Egypt; 2 Department of Chemistry, Faculty of Education, Ain Shams University, Roxy, Cairo, 11757 Egypt

**Keywords:** Semicarbazone, Pyrano[3,2-c]quinoline, Copper complex, DFT, NLO, NBO, Chemical biology, Chemistry

## Abstract

A novel Cu(II) complex was synthesized from a reaction of 2-[(6-ethyl-4-hydroxy-2,5-dioxo-5,6-dihydro-2H-pyrano[3,2-c]quinolin-3-yl)methylidene]hydrazinecarboxamide (PQMHC) ligand with copper sulfate in a molar ratio 1:1 (L:M). Cu(II)-PQMHC complex was characterized by elemental analysis, mass spectra, infrared spectra, molar conductance, electron spin resonance spectra, electronic spectra, and thermal analysis. Kinetic parameters for the thermal decomposition steps were evaluated using the Coats–Redfern equations. The results of spectral data revealed that the ligand behaves as an O_2_N tridentate donor, coordinating through the hydroxyl group, azomethine nitrogen, and keto oxygen, forming a square planar geometry. Based on DFT, the molecular structure was optimized at B3LYP/6-311G(d, P) for the PQMHC ligand and at B3LYP/GENECP for the Cu(II)-PQMHC complex and interrelated with the experimental results. The optimized geometry of the Cu(II)-PQMHC complex is nonplanner as indicated by the dihedral angles. The HOMO–LUMO energy levels, total dipole moment, molecular electrostatic potential (MEP), thermodynamic properties, natural population analysis (NPA), natural bond orbital (NBO) analysis, non-linear optical (NLO) behavior, along with both global and local chemical reactivity descriptors were investigated. The electronic absorption spectra of the PQMHC ligand and its copper complex were investigated through the time-dependent density functional theory (TD-DFT) method at the CAM-B3LYP.

**Supplementary Information:**

The online version contains supplementary material available at 10.1038/s41598-025-26205-8.

## Introduction

Pyrano[3,2-c]quinolines are part of an important class of nitrogen heterocycles that have been widely used in the pharmaceutical industry^1,2^. They are found in numerous natural alkaloids^3^, and exhibit diverse pharmacological activities such as antimicrobial^4^, antitubercular^5^, anti-diabetic ^6^, antihypertensive^7^, antitumor^8^, anti-coronavirus^9^, antimalarial^10^, as well as thin films^11^, optical applications^12^, and corrosion inhibitors^13^. According to previous reports, semicarbazones have a variety of structural characteristics, and their metal complexes exhibit greater biological activity than their free ligands^14^. Different coordination modes between semicarbazones and metal ions are influenced by a variety of parameters, including the type of counter ions, the metal salt, the substituent on the carbonyl function, and the reaction conditions^15^. Additionally, by complexing with certain metal ions, semicarbazones increase the bioactivity of existing medications, enabling the development of new and more effective drugs. In addition to their analytical uses, semicarbazone transition metal complexes have been the subject of much research because of their coordination capabilities^16^.

Furthermore, density functional theory (DFT) is a widely accepted and reliable computational method by the global scientific community, extensively employed to predict the electronic structures, geometries, and reactivity of transition metal complexes^17,18^. For the PQMHC ligand, the use of B3LYP^19^ with the 6-311G(d, P) basis set has proven effective in accurately reproducing geometric configurations, vibrational behaviors, and electronic attributes in agreement with experimental results. This effectiveness is attributed to the PQMHC ligand’s large, complex organic structure, which features multiple functional groups such as aromatic rings, nitrogen, and oxygen atoms. The Gen keyword in Gaussian allows users to define a custom basis set for their calculations, replacing the standard basis set or density fitting basis set keywords. When using Gen, the basis set details must be manually provided in a separate input section. Similarly, Gen can be applied to specify a different density fitting basis set. The Gen ECP option enables the input of both basis functions and effective core potentials (ECPs), functioning the same way as Gen Pseudo = Read. The LANL2DZ (Los Alamos National Laboratory 2 Double-Zeta) basis set, a commonly used effective core potential (ECP) basis set, was employed to model the metal atoms. This type of mixed basis set has been widely applied alongside density functional methods in the study of systems containing transition metals. In recent years, such mixed basis sets have gained significant popularity in computational chemistry research within this field^20^. The performance of the mixed 6-311G(d, P) + LANL2DZ basis set was specifically assessed for the Cu(II)-PQMHC complex. It provides valuable support for interpreting and elucidating the detailed molecular structures and associated characteristics. The geometrical parameters were calculated using B3LYP/GENECP/6-311G(d,P) basis set. The time-dependent density functional theory (TD-DFT) calculations were performed to determine the energies of the excited states and the spectra of the electronic absorption, utilizing the Coulomb-attenuating system CAM-B3LYP hybrid exchange–correlation functional^21^. Recognizing the deficiencies of the conventional B3LYP functional in modeling excited states, CAM-B3LYP was specifically designed to incorporate a Coulomb-attenuating scheme, thereby enhancing the description of long-range electron–electron interactions^22^.

A number of studies have highlighted the structural versatility, coordination chemistry, and bioactivity of thiosemicarbazone- and Schiff base–derived metal complexes. Jain et al*.*^23^ synthesized and structurally characterized Ni(II) and Cu(II) complexes of a bidentate thiosemicarbazone ligand, integrating theoretical calculations and molecular modeling to correlate geometry optimization results with experimental data. Their findings demonstrated significant antibacterial activity, particularly for the Cu(II) complex, which was attributed to enhanced chelation and improved lipophilicity. Extending this line of research, Jain et al*.*^24^ reported the synthesis of bioactive thiosemicarbazone coordination metal complexes, incorporating in-depth characterization, DFT-based theoretical studies, molecular docking, and ADME predictions. Their work revealed promising anticancer and antimicrobial properties, with docking results indicating strong interactions with biologically relevant enzymes, thereby validating the pharmacological potential of these complexes.

Earlier, Goel et al*.*^25^ investigated the synthesis and spectroscopic characterization of a tridentate Schiff base ligand—2-acetyl-5-methyl-furanthiosemicarbazone—and its Mn(II), Co(II), Ni(II), and Cu(II) complexes. Their spectral studies confirmed tridentate coordination via nitrogen, oxygen, and sulfur donor atoms, while biological screening revealed notable antimicrobial efficacy, particularly for the Ni(II) complex. In subsequent work, Goel et al*.*^26^ designed methylcarbamatethiosemicarbazone derivatives and their Mn(II) and Co(II) complexes, performing antibacterial assays that demonstrated improved activity for the metal complexes over the free ligand. Molecular modeling further supported these results, showing favorable binding orientations and interaction energies with microbial targets.

Complementing these studies, Gandhi et al*.*^27^ presented a comprehensive review on quinoline Schiff bases (QSBs) and their derivatives, focusing on their emerging role as antimicrobial agents. The review underscored how structural modifications, including electron-donating and electron-withdrawing substituents, influence biological activity, and emphasized the potential of QSB-based frameworks for designing next-generation antimicrobial therapeutics. Collectively, these studies establish that rational ligand design, coupled with appropriate metal coordination, can significantly enhance biological performance, supporting the development of multifunctional coordination compounds with therapeutic and material applications.

Due to our ongoing curiosity in metal complexes derived from pyrano[3,2-c]quinoline-3-carboxaldehyde and its semicarbazone ligands^28–31^, the current study focuses on the synthesis of a Cu(II)-PQMHC complex, by reacting pyrano[3,2-c]quinoline-3-carboxaldehyde-semicarbazone ligand (PQMHC) with copper sulfate in a 1:1 molar ratio. The structure of the resulting complex was elucidated through elemental analysis, molar conductance measurements, spectral studies, and thermogravimetric analysis.

Although pyranoquinoline-based semicarbazones and their transition metal complexes have been explored for biological and analytical applications, a systematic study integrating experimental synthesis, spectroscopic characterization, and advanced computational modeling, particularly with a mixed B3LYP/6-311G(d,p) + LANL2DZ approach, remains largely unexplored. Previous works have focused on either biological evaluation or basic structural characterization, leaving a gap in correlating detailed quantum chemical insights with potential optoelectronic and biomedical applications. The present work addresses this gap by synthesizing and characterizing a novel Cu(II)–PQMHC complex, performing comprehensive spectroscopic analysis, and applying state-of-the-art computational methods to elucidate its structural, electronic, NLO, and excited-state properties. This integrated approach provides not only a deeper understanding of the structure–property relationship but also identifies the compound’s potential in multifunctional applications spanning biomedical and optical domains.

This study aims to deliver a thorough theoretical analysis using density functional theory (DFT) for a newly developed and carefully synthesized PQMHC ligand and its Cu(II)-PQMHC complex, both of which display semiconducting properties. It represents the first detailed DFT examination of the PQMHC ligand and its Cu(II) complex. Through DFT calculations, our findings are anticipated to greatly enhance the understanding of their molecular structures, electronic characteristics, and optical spectra. The global reactivity descriptors of the free ligand and Cu(II)-PQMHC complex were calculated and analyzed. In addition, the NLO of the compounds under study has been investigated. Furthermore, Natural Bond Orbital (NBO) analysis was carried out to obtain the charge distribution and precise electronic configuration of the active centers within the ligands and the Cu metal in the studied complex.

## Experimental

### Materials

The chemical CuSO_4_.5H_2_O was employed as BDH. Disodium ethylenediaminetetraacetic acid (EDTA) salt, nitric acid, ammonium hydroxide, and murexide were obtained from either BDH or Merck. Reagent-grade organic solvents (ethanol, diethyl ether, dimethylformamide [DMF]) were employed throughout the study without additional purification.

### Synthesis of the H_2_L ligand

**Scheme 1:**
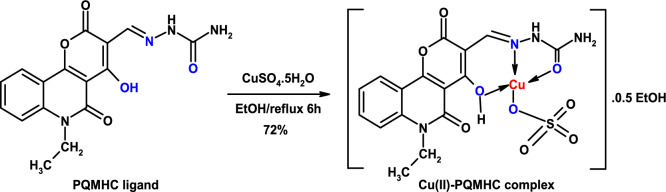
Synthesis and proposed structure of the Cu(II)-PQMHC complex.

As cited in the literature, the 2-[(6-ethyl-4-hydroxy-2,5-dioxo-5,6-dihydro-2H-pyrano[3,2-c]quinolin-3-yl)methylidene]hydrazinecarboxamide (PQMHC) ligand (Scheme 1) was prepared^30^.

### Synthesis of the Cu(II)-PQMHC complex

An aqueous solution of LiOH·H_2_O (0.08 g, 2.00 mmol in 5 mL water) was added dropwise with stirring maintained throughout to a hot solution of the H_2_L ligand (0.52 g, 2.00 mmol). CuSO_4_.5H_2_O (0.59 g, 2.00 mmol in 20 mL of ethanol was gradually added under continuous stirring, providing a 1:1 molar ratio. Refluxing the reaction mixture for 6 h led to the formation of a yellow solid. The solid was then filtered, washed with ethanol and diethyl ether, and finally air-dried. The obtained yield (Scheme 1) was 0.76 g (72%). This proposed structure was confirmed using spectroscopic methods as discussed below.

### Physical measurements

The melting points of the synthesized compounds were determined using a Stuart SMP3 melting point apparatus. Elemental analyses (C, H, N, and S) were conducted with a Vario EL-Elementar analyzer. Determination of metal content involved acid digestion of the complexes with concentrated nitric acid, followed by complexometric titration using EDTA. Mass spectra and mass fragmentations were recorded using a Shimadzu Gas chromatography GC-2010 instrument mass spectrometer (70 eV). Fourier-transform infrared (FTIR) spectra were recorded using a Nicolet IS10 spectrometer with KBr pellets. The electronic absorption spectra were obtained using a JASCO V-550 UV/Vis spectrophotometer at ambient temperature, utilizing solid-state reflectance, and/or DMF solutions. A Johnson Matthey balance (Model MKI, Alfa Product) was used to determine the magnetic susceptibility of the complex. Effective magnetic moments were determined and adjusted for diamagnetic contributions using Pascal’s constants. Molar conductivity was measured using a Corning conductivity meter (Model 441, NY 14,831) for 10^–3^ M solutions of the solid complex in DMF. A Bruker Elexsys E500 spectrometer (Germany) was employed to record the electron spin resonance (ESR) spectra of the complex. A 4.7 mg sample was subjected to thermogravimetric analysis on a Shimadzu-50H, heating from ambient temperature to 1000  °C at 10 °C/min.

### Computational methods

All computational procedures were carried out using the Gaussian 09W software package^32^, and molecular visualizations were prepared with GaussView 5.0.9^33^. Density Functional Theory (DFT) calculations for the PQMHC ligand were performed with the B3LYP functional in combination with the 6-311G(d, P) basis set. For the Cu(II)–PQMHC complex, the B3LYP/GENECP method was employed, applying the 6-311G(d, P) basis set to C, H, N, O, and S atoms, and the LANL2DZ effective core potential basis set to the copper center. The choice of B3LYP with the mixed 6-311G(d, P)/LANL2DZ basis set was made due to its established accuracy and computational efficiency in modeling transition-metal complexes, particularly those involving Cu(II). Literature comparisons indicate that B3LYP often yields optimized geometries, vibrational modes, and electronic properties in close agreement with experimental data, performing comparably to functionals such as M06 and PBE0, but at a lower computational cost. This validation supports its suitability for the present system.

Ground-state geometries of both the PQMHC ligand and Cu(II)–PQMHC complex were fully optimized, followed by vibrational frequency analyses to confirm all stationary points as true minima. A broad range of physical and electronic properties was evaluated, including thermochemical parameters, HOMO–LUMO energy gap, total dipole moment (TDM), nuclear repulsion energy, ionization energy (I), electron affinity (A), global electrophilicity index (ω), electronic chemical potential (V), global hardness (η), electronegativity (χ), and softness (S). Non-quantum global reactivity descriptors were computed using HyperChem 8.0.10^34^. Nonlinear optical (NLO) parameters—total static dipole moment (μ), mean polarizability (α), polarizability anisotropy (Δα), and mean first-order hyperpolarizability (β)—were determined at the same theoretical level, along with second-order NLO properties such as hyper-Rayleigh scattering (β_HRS_) and depolarization ratio (DR).

Natural Bond Orbital (NBO) analyses were conducted using the NBO 3.1 program^35^ as implemented in Gaussian 09W, while density of states (DOS) spectra were obtained using GaussSum 3.0^36^. Excited-state energies and electronic absorption spectra were calculated in both the gas phase and DMF solvent using the SCRF approach with the Integral Equation Formalism Polarizable Continuum Model (IEFPCM)^37^, employing TD-DFT at the CAM-B3LYP level with 20 vertical excitation states. This functional was chosen for excited-state calculations because of its improved treatment of long-range electron–electron interactions compared to conventional B3LYP. Finally, thermodynamic functions under ideal gas conditions were calculated using the THERMO PL Perl-based script^38^.

## Results and discussion

### Elemental analysis and mass spectra

**Fig. 1:**
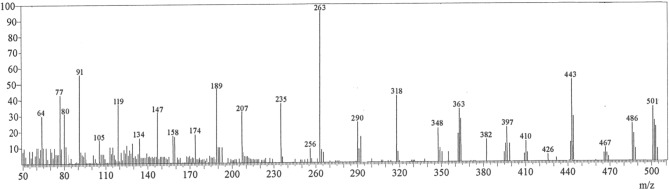
Mass spectrum of Cu(II)-PQMHC complex.

**Scheme 2:**
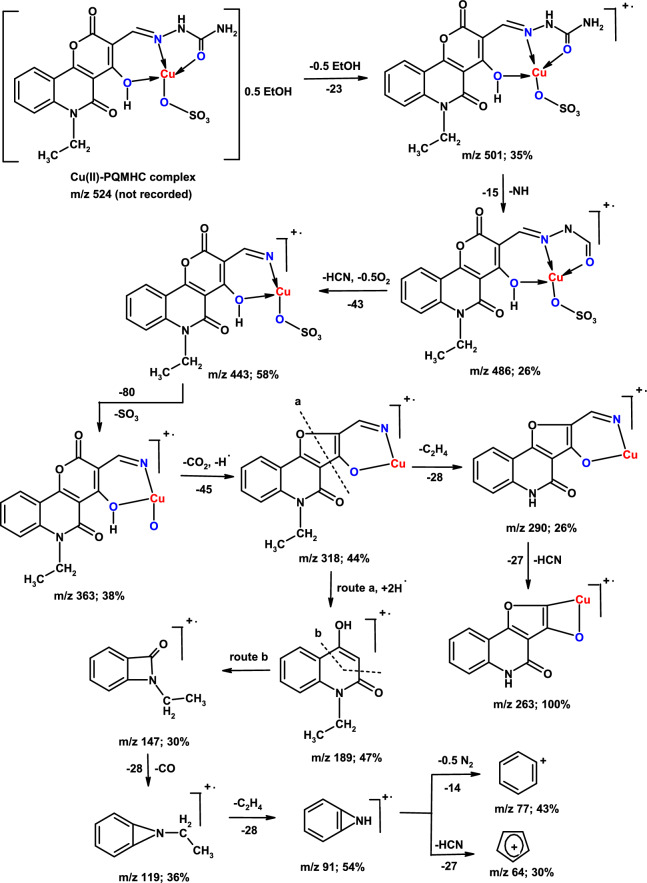
Mass fragmentation pattern of Cu(II)-PQMHC complex.

The synthesized copper complex is non-hygroscopic, stable at ambient temperature, and exhibits limited solubility in water and popular organic solvents. Table 1 illustrates the key analytical and physical data of the PQMHC ligand and its Cu(II) complex, including their molecular formula (M. F.), molecular weight (F. Wt.), elemental analyses, and melting points (M. P.). Mass spectrometry constitutes a powerful analytical tool for the verification of proposed molecular structures. The mass spectrum and corresponding fragmentation patterns of the PQMHC ligand have been reported and analyzed in the literature^30^. The mass spectrum of the PQMHC ligand revealed the presence of the parent ion peak alongside multiple fragment peaks. Figure 1 presents the mass spectrum of the Cu(II)-PQMHC complex with its fragmentation patterns illustrated in Scheme 2. The molecular ion peak observed at *m/z* 501 corresponds closely to the molecular weight (524.95), which is in good agreement with the proposed formula weight of the anhydrous complex [Cu (H_2_L)(SO_4_)] (F. Wt. = 501.92) suggested by elemental analysis (Table 1).

**Table 1:** Analytical and physical data of the PQMHC ligand and its Copper (II) complex.

Reaction	M. F [F. Wt.]	Abbreviation	Color	Yield g (%)	M.P. ºC	Elemental analysis, % found/(calc.)			
						C	H	N	S
H_2_L	C_16_H_14_N_4_O_5_ [342.31]	PQMHC	Yellow	2.48 (73)	220	56.59 (56.14)	4.10 (4.12)	16.33 (16.37)	–
H_2_L + CuSO_4_.5H_2_O	[Cu (H_2_L)(SO_4_)].0.5EtOH C_16_H_14_N_4_O_9_SCu [501.92]	Cu(II)-PQMHC	Yellow	0.76 (72)	260	38.15 (38.29)	2.70 (2.81)	11.13 (11.16)	6.28 (6.39)

### IR spectra

**Fig. 2:**
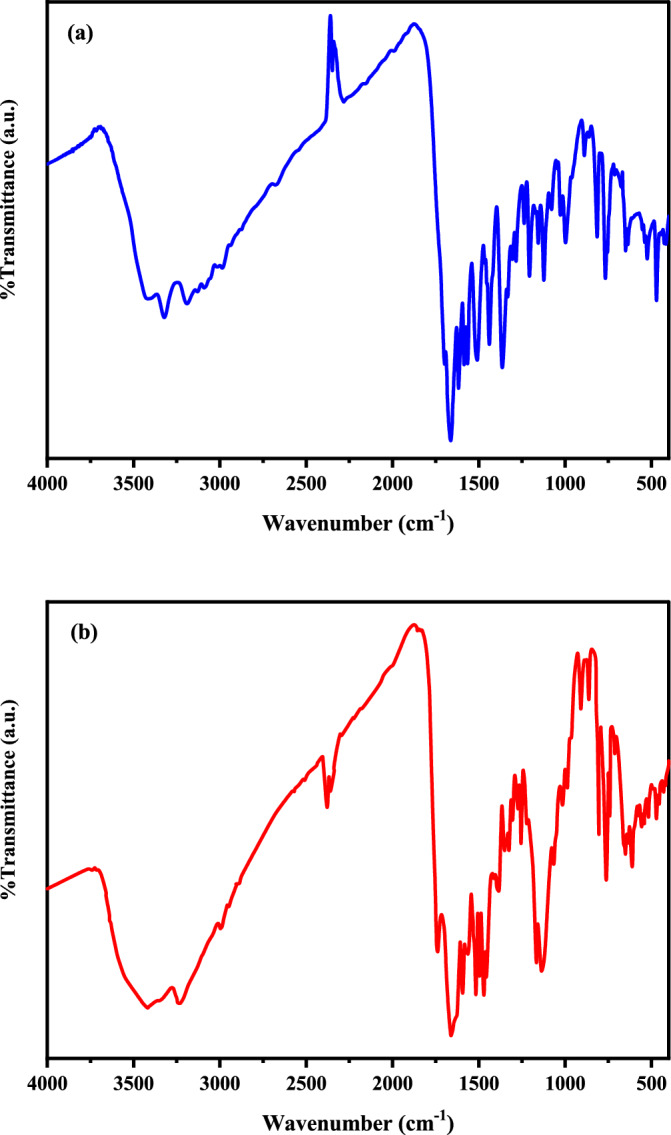
Experimental IR spectra for **a** PQMHC ligand and **b** Cu(II)-PQMHC complex.

The IR spectra of the PQMHC ligand and its Cu(II)-PQMHC complex were recorded in the 4000–400 cm⁻^1^ region using KBr pellets, as shown in Fig. 2a and b. The main vibrational bands with their corresponding assignments are summarized in Table 2. A comparison between the spectra of the free ligand and the Cu(II) complex provides insight into the coordination behavior^39^. The C=N stretching vibration, observed at 1586 cm⁻^1^ in the ligand, shifts to 1582 cm⁻^1^ upon complexation, confirming the coordination of the azomethine nitrogen ^40^. In the complex, a broad absorption at 3416 cm⁻^1^ can be ascribed to O–H stretching from the ligand and the presence of a non-coordinated ethanol molecule. The strong band at 1663 cm⁻^1^ in the ligand, assigned to the amide C=O group, appears at 1651 cm⁻^1^ in the complex, supporting its participation in bonding in the keto–amine tautomeric form (Scheme 1)^41^. Furthermore, the broad band at 3231 cm⁻^1^ corresponds to N–H stretching modes, confirming that the PQMHC ligand remains in the keto tautomeric state during complex formation. The coordination of the sulfate group is evidenced by the characteristic bands at 1134 and 1062 cm⁻^1^, which are consistent with a monodentate sulfato mode^42^. In addition, the weak absorptions observed near 475 cm⁻^1^ and 563 cm⁻^1^ are attributed to Cu–O and Cu–N stretching vibrations, respectively^43^. These spectral features collectively provide strong support for the proposed tautomeric behavior of the ligand and the coordination of sulfate in the complex^42^.

**Table 2:** Typical infrared (IR) spectral data of the PQMHC ligand and its Copper (II) complex.

Compound	IR Spectra (cm^−1^)									
	ν(OH) H_2_O/EtOH	ν(NH_2_)	ν(NH)	ν(OC = O)	ν(C=O) amide	ν(C=O) quinolinone	ν(C=N) chelated	ν(Cu–N)	ν(Cu–O)	Other bands
PQMHC	3449 s	3322, 3191	3133	1729 m	1663	1617 s	1586 s	–	–	–
Cu(II)-PQMHC	3416 br	3231	3231	1724 m	1651 sh	1619 sh	1582 m	563 w	475 w	1134, 1062; (SO_4_) (monodentate)

*br* broad band, *m* medium band, *s* strong band, *sh* shoulder band, *w* weak band.

### Electronic spectra and magnetic measurements

**Fig. 3:**
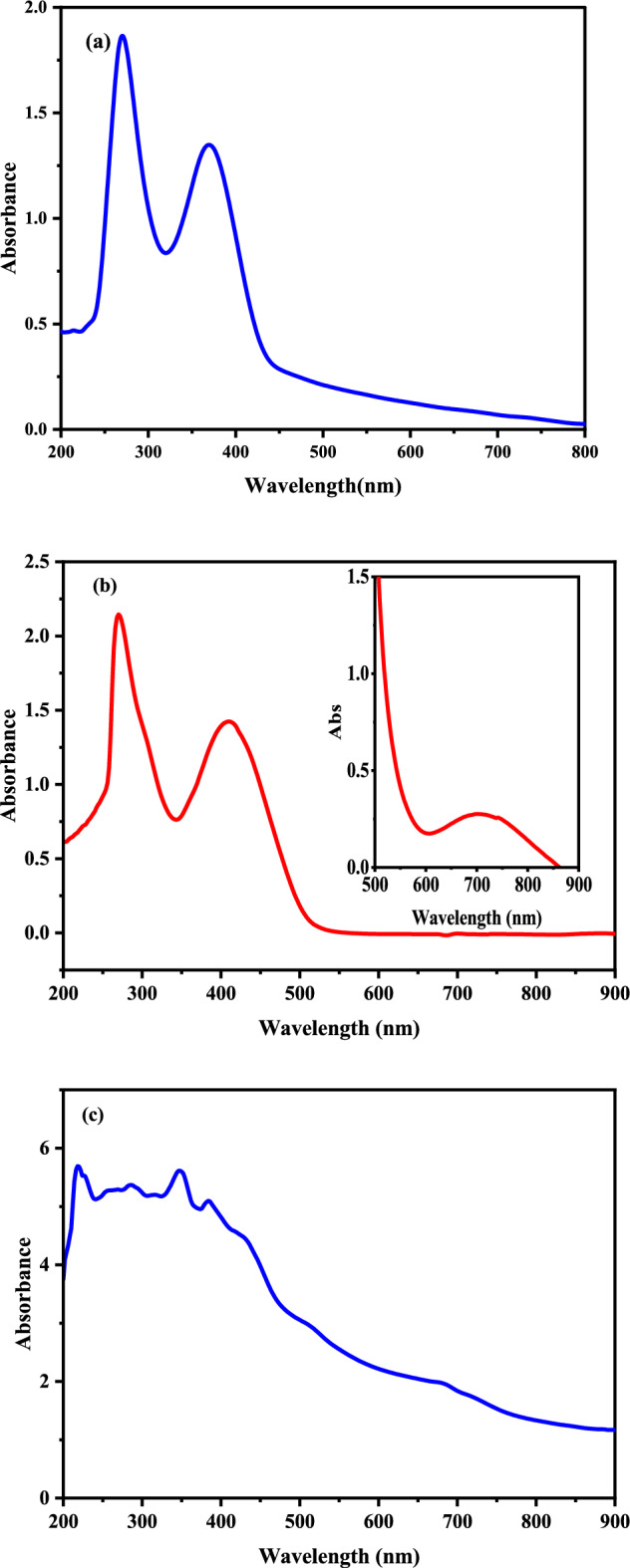
Experimental UV–Vis spectra of **a** PQMHC ligand in DMF, **b** Cu(II)-PQMHC complex in DMF (the inset in concentrated solution), and **c** Cu(II)-PQMHC complex in reflectance.

Upon detailed comparison of the electronic spectra of the PQMHC ligand and its Cu(II) complex, band shifts toward the red or blue regions were observed, confirming complex formation. Additionally, new bands appeared in the spectra of the complexes, as summarized in Table 3. Figure 3a illustrates the electronic spectrum of the PQMHC ligand in DMF, displaying two absorption bands: one at 270 nm, attributed to the π–π* quinolone ring-centered electronic transition, and another at 369 nm, corresponding to the n-π* transition of the side-chain amide functional group^30^. The electronic spectra data of Cu(II)-PQMHC complex in DMF and reflectance are displayed in Fig. 3b and c, respectively.

**Table 3:** Electronic spectrum, magnetic moment and molar conductivity data of the PQMHC ligand and its Copper (II) complex.

Compound	Electronic spectral bands (nm) λ_max_ (nm)/(ε_max_ L cm^–1^ mol^–1^) DMF (reflectance)				μ_eff_ B.M	Conductance (Ω^–1^ cm^2^ mol^–1^)	Geometry
	π–π* quinolinone	n–π* amide	d–d transition	Assignment			
PQMHC	270	369	–	–	–	–	–
Cu(II)-PQMHC	270 (284)	368 (348)	410, 704 (434, 700)	^2^B_1_ ← ^2^B_2_	1.68	15.20	Square planar

The electronic spectrum of the d⁹ Cu(II)–PQMHC complex, recorded in DMF solution (reflectance), exhibits four distinct absorption bands. The two higher energy bands, observed at 270 nm (284 nm) and 368 nm (348 nm), fall within the characteristic ligand-centered (π–π* or n–π*) transition region, indicative of transitions within the ligand framework itself. In contrast, the lower-energy absorptions at 410 nm (434 nm) and 704 nm (700 nm) are attributed to ligand-to-metal charge transfer (LMCT) and d–d transitions, respectively. Specifically, the band near 704 nm corresponds to the spin-allowed d–d transition from the d_xy_ to the d_x_^2^_–y_^2^ orbital (^2^B₁ ← ^2^B₂), consistent with a square planar ligand field around the Cu(II) center. This assignment aligns with ligand field theory, where in a square planar crystal field, the d-orbital energies are significantly split, with the d_x_^2^_–y_^2^ orbital lying highest in energy due to its strong σ-antibonding interactions with the ligand in the xy-plane. The observed spectral features, particularly the low-energy d–d transition, support a distorted square planar geometry, commonly observed in d⁹ Cu(II) complexes due to the Jahn–Teller effect^42,44^. In such systems, the degeneracy of the eg orbitals (d_x_^2^_–y_^2^ and d_z_^2^) is lifted, leading to elongation or compression along one axis, typically resulting in a tetragonally distorted square planar or octahedral geometry. In this case, the spectroscopic data suggest significant distortion, favoring a square planar configuration as the predominant geometry^45^. Furthermore, the magnetic moment of the complex was determined to be 1.68 B.M., which is in good agreement with the theoretical spin-only value (1.73 B.M.) expected for a single unpaired electron (S = ½) in a d⁹ configuration. This magnetic data further corroborates the presence of a single unpaired electron residing in the d_x_^2^_–y_^2^ orbital, again consistent with a square planar geometry imposed by a strong ligand field and stabilized by the Jahn–Teller distortion. Altogether, these spectroscopic and magnetic observations reinforce the assignment of a distorted square planar geometry around the Cu(II) center in the complex.

### Conductivity measurements and ESR spectrum

The molar conductivity value was determined at ambient temperature in a 10^–3^ M DMF solution. The Cu(II)-PQMHC complex was found to be non-electrolytic. Its molar conductance of 15.20 Ω^-1^ cm^2^ mol^-1^ supports a non-electrolytic nature, indicating that the sulphate anion is situated inside the metal coordination sphere^43,46^.

**Fig. 4:**
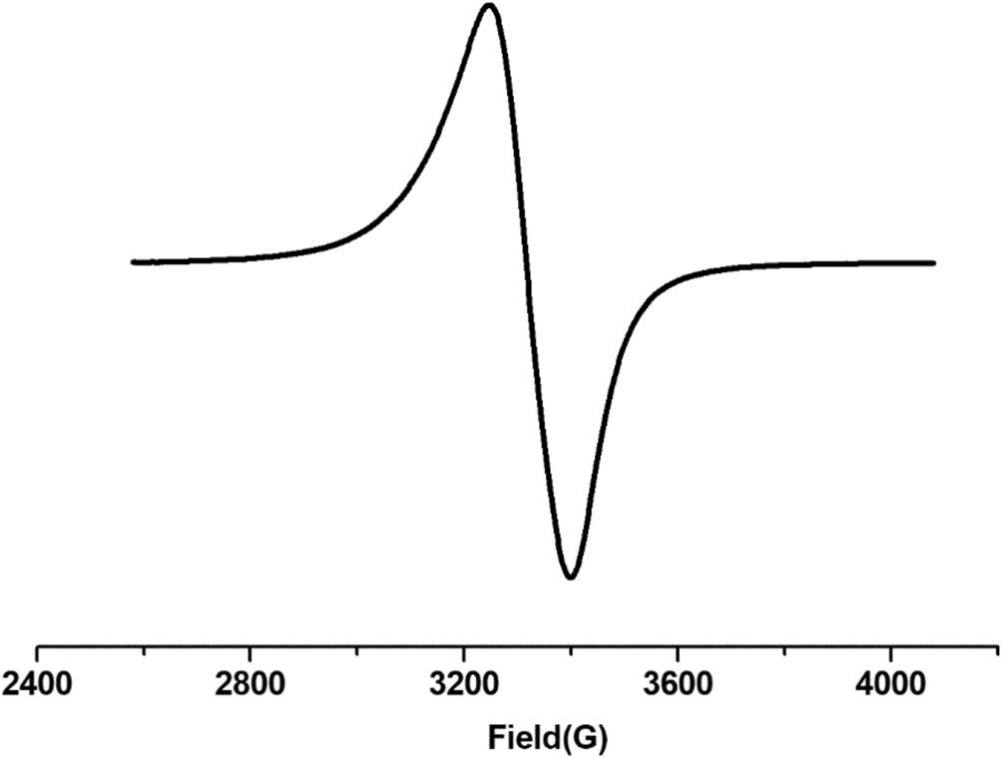
X-band ESR spectrum of Cu(II)-PQMHC complex.

Figure 4 illustrates the solid-state electron spin resonance (ESR) spectrum of the Cu(II)-PQMHC complex acquired at ambient temperature. The Cu(II)-PQMHC complex displays one broad band with g_eff_ = 2.09, and the spectrum’s shape confirms that the complex is square planar geometry^41,47^.

The electron spin resonance (ESR) spectrum of the Cu(II)-PQMHC complex was recorded in a solid state at room temperature, as shown in Fig. 4. The spectrum displays a single broad signal with an effective g-value (g_eff_) of 2.09. The absence of well-resolved hyperfine splitting, along with the shape and broadness of the band, suggests a strong interaction between the unpaired electron and the surrounding ligand field. The observed g_eff_ value, which is slightly higher than the free electron value (g_e_ = 2.0023), is consistent with the presence of a Cu(II) ion (d^9^, S = 1/2) in a square planar environment. In such geometries, the unpaired electron typically resides in the d_x_^2^_–y_^2^ orbital, which lies in the molecular plane and experiences strong ligand field interactions, leading to axial-type spectra with small g-anisotropy^41,47^.

The electron spin resonance (ESR) spectrum of the Cu(II)-PQMHC complex was recorded in a solid state at room temperature, as shown in Fig. 4. The spectrum displays a single broad signal with an effective g-value (g_eff_) of 2.09. The absence of well-resolved hyperfine splitting, along with the shape and broadness of the band, suggests a strong interaction between the unpaired electron and the surrounding ligand field. The observed g_eff_ value, which is slightly higher than the free electron value (g_e_ = 2.0023), is consistent with the presence of a Cu(II) ion (d^9^, S = 1/2) in a square planar environment. In such geometries, the unpaired electron typically resides in the d_x_^2^_–y_^2^ orbital, which lies in the molecular plane and experiences strong ligand field interactions, leading to axial-type spectra with small g-anisotropy.

### Thermogravimetric analysis

**Fig. 5:**
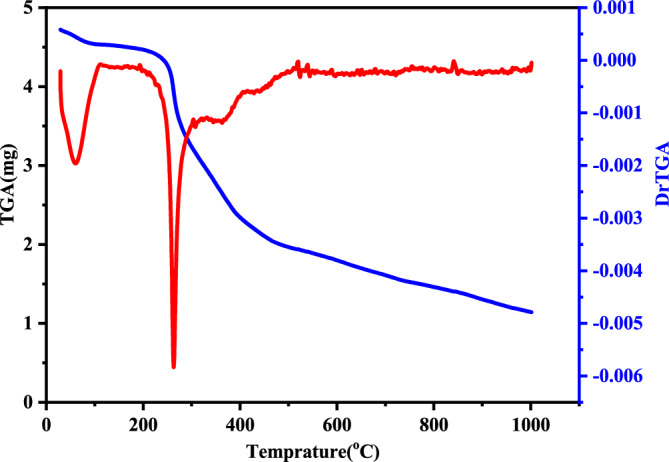
TGA-DrTGA curves of Cu(II)-PQMHC complex.

To assess the thermal stability of the complex and to distinguish between coordinated and lattice solvent molecules, thermogravimetric analysis (TGA) together with differential thermogravimetric (DTG) measurements was carried out over the temperature range from room temperature up to 900 °C. The TGA curve of the Cu(II)–PQMHC complex (Fig. 5, Table 4, and Table S1) indicates a decomposition process that proceeds through three distinct stages. The first step occurs between 30 and 148 °C, corresponding to the loss of half a molecule of uncoordinated ethanol, released at approximately 60 °C. The experimental mass loss of 4.27% aligns closely with the calculated value of 4.38%, confirming the assignment. The second step, observed between 148 and 300 °C, is attributed to the elimination of one H₂SO₄ molecule, with decomposition peaking at 264 °C. The weight loss measured (18.98%) is in excellent agreement with the calculated value (18.70%). The final decomposition stage, occurring between 300 and 1000 °C with a maximum at 445 °C, involves the loss of the C₁₂H₉N₄O₄ organic moiety, resulting in an observed mass loss of 51.58% (calculated: 52.09%). The residue corresponds to CuO and C₄H₄, with observed and calculated values of 24.62% and 25.10%, respectively.

**Table 4:** Temperatures of decomposition and the kinetic parameters of the Copper (II)-PQMHC complex.

Complex	Step	N order	T (K)	A (S^-1^)	E_a_ (kJ mol^-1^)	ΔH (kJ mol^-1^)	ΔS (kJ mol^1^ K^-1^)	ΔG (kJ mol^-1^)
Cu(II)-PQMHC	1st	1	343	12.42 × 10^4^	32.43	29.57	– 0.156	83.08
	2nd	1	533	15.48 × 10^4^	88.24	83.80	– 0.104	138.97
	3rd	1	723	24.33 × 10^7^	16.87	10.86	– 0.094	78.83

Thermal kinetic parameters—namely enthalpy (ΔH), entropy (ΔS), Gibbs free energy (ΔG), and activation energy (E_a_) were determined for each decomposition step using the Coats–Redfern integral method^48,49^ (Table 4). The positive values of ΔH confirm that the decomposition process is endothermic. In addition, the lower E_a_ observed in the second stage compared to the first indicates a higher decomposition rate^50^. The negative ΔS values reflect the formation of a more ordered activated complex relative to the reactants, implying reduced molecular randomness in the transition state and a comparatively slower reaction rate. Furthermore, the slightly positive ΔG values suggest that the processes are non-spontaneous, supporting the view that the thermal decomposition is influenced by the autocatalytic effect of the metal ions^50^.

### Ground state geometry optimization

**Fig. 6:**
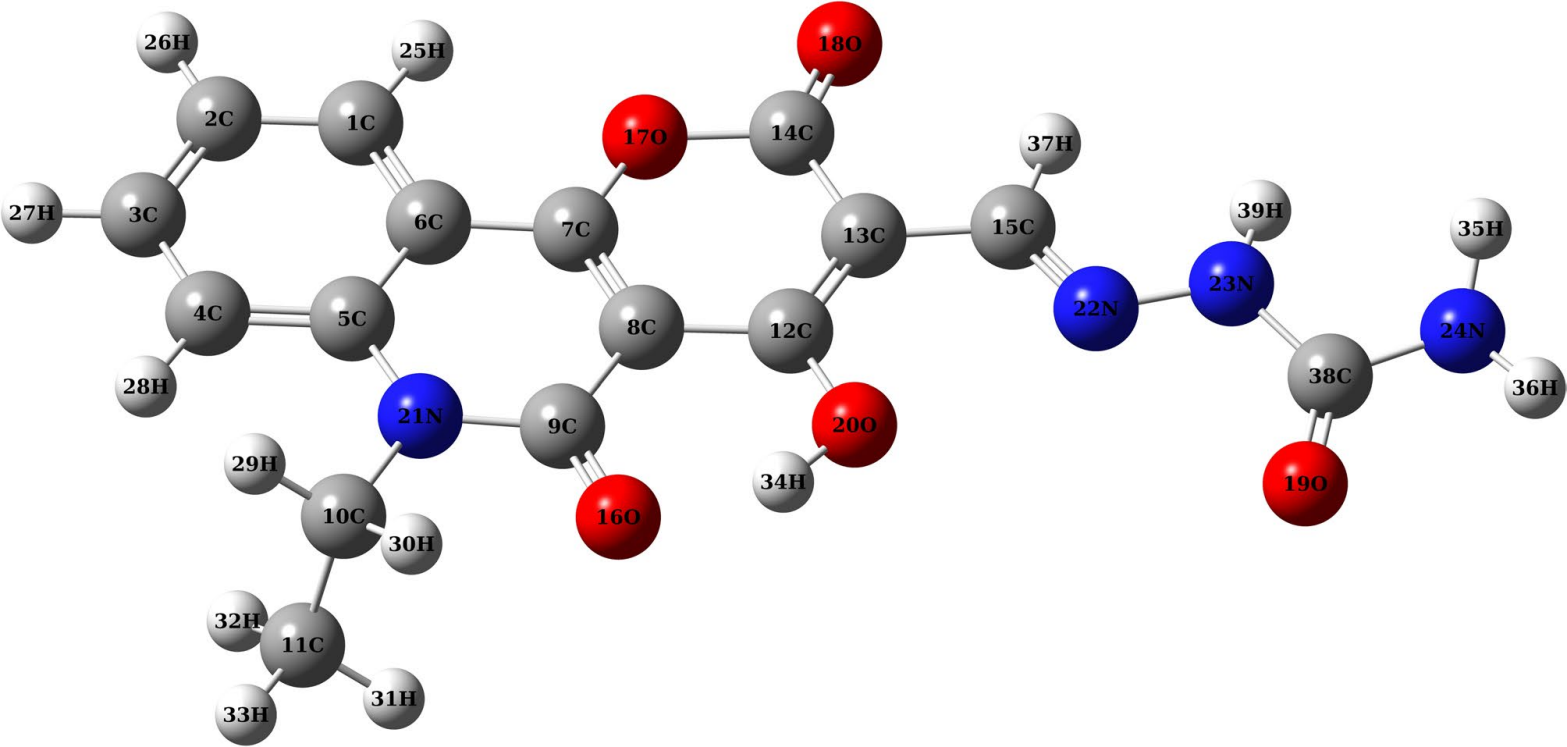
Optimized geometry of the PQMHC ligand using B3LYP/6-311G(d,P).

**Fig. 7:**
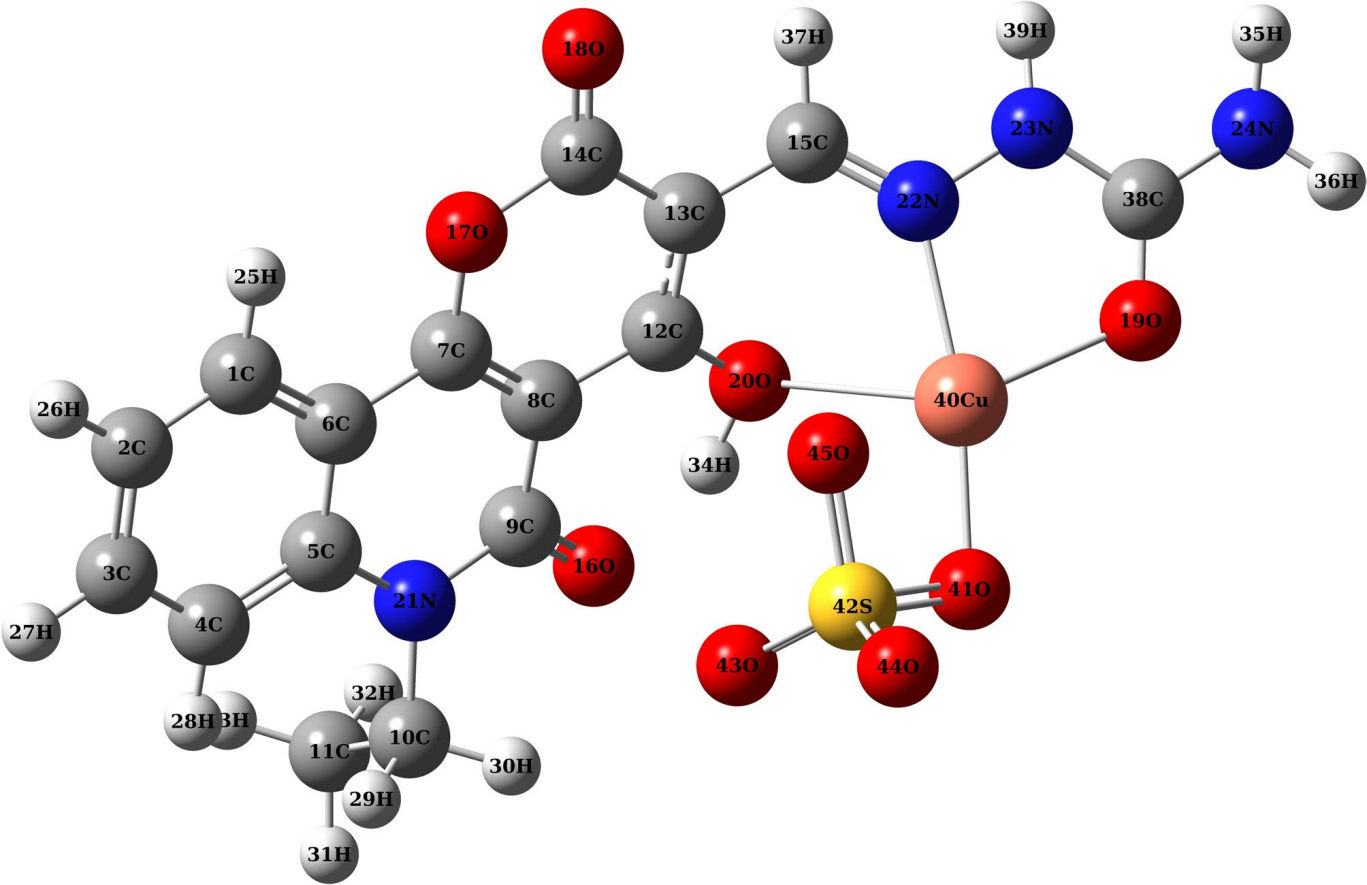
Optimized geometry of the Copper (II)-PQMHC complex using B3LYP/GENECP.

The optimized ground state molecular structures and atom numbering scheme of the PQMHC ligand (B3LYP/6-311G(d, P)), and its copper(II) complex (B3LYP/GENECP), are shown in Figs. 6 and 7, respectively. The vibrational analysis carried out at the same theoretical level verified that the optimized geometries represent true minima, since no imaginary frequencies were detected. A summary of the key structural parameters, including selected bond lengths, bond angles, and dihedral angles for both compounds, is provided in Table 5. For the PQMHC ligand, all calculated bond lengths and bond angles are consistent with values reported in the literature^22^. Examination of the dihedral angles indicates that the PQMHC ligand is generally planar, except for the C11C10N21C9 (-93.16°) and O19C38N24H35 (– 146.25°) dihedral angles, which deviate significantly from the molecular plane. This geometry likely corresponds to the most stable conformation.

**Table 5:** Selected equilibrium bond lengths, (Å), bond angles, (°) and dihedral angles, (°) for the PQMHC ligand computed at the B3LYP/6‐311G (d,P), and its Copper (II) complex computed at the B3LYP/GENECP.

Bond lengths (Å)	PQMHC	Cu(II)-PQMHC	Bond angles (°)	PQMHC	Cu(II)-PQMHC	Dihedral angles (°)	PQMHC	Cu(II)-PQMHC
N21-C5	1.396	1.393	< C5N21C9	122.64	122.12	< O18C14O17C7	– 179.82	178.11
N21-C9	1.388	1.379	< C5N21C10	121.24	122.05	< O16C9N21C5	– 177.84	– 171.31
N21-C10	1.479	1.481	< C9N21C10	116.11	115.81	< C11C10N21C9	– 93.16	– 91.97
O16-C9	1.242	1.241	< O16C9N21	120.23	121.23	< O20C12C13C15	0.23	– 6.73
O17-C7	1.334	1.340	< O16C9C8	122.00	121.40	< C12C13C15N22	0.62	– 27.58
O17-C14	1.431	1.423	< C7O17C14	122.68	122.40	< C13C15N22N23	– 179.16	176.36
O18-C14	1.201	1.200	< O17C14O18	115.19	116.41	< O19C38N24H35	– 146.25	– 162.72
O20-C12	1.317	1.318	< O17C14C13	116.63	116.15	< C12O20Cu40O41	–	110.86
O20-H34	0.993	1.017	< O18C14C13	128.18	127.44	< C12O20Cu40N22	–	– 65.48
N22-C15	1.283	1.289	< H34O20C12	107.47	106.51	< C12O20Cu40O19	–	– 128.76
N22-N23	1.353	1.377	< O20C12C8	119.55	119.02	< O20Cu40O41S42	–	– 75.23
N23-C38	1.397	1.380	< O20C12C13	120.92	121.76	< N22Cu40O41S42	–	– 54.55
O19-C38	1.206	1.235	< N22C15C13	124.43	122.08	< Cu40O41S42O44	–	– 118.53
N24-C38	1.395	1.356	< N22C15H37	121.55	120.82	< Cu40O41S42O43	–	109.32
N24-H35	1.010	1.007	< N23N22C15	116.03	119.76			
N24-H36	1.009	1.008	< N22N23C38	120.58	114.94			
Cu40-O19	–-	2.106	< N23C38O19	124.90	120.90			
Cu40-O20	–-	2.620	< O19C38N24	123.81	122.61			
Cu40-N22	–-	2.078	< N23C38N24	111.26	116.49			
Cu40-O41	–-	1.929	< O19Cu40N22	–-	77.10			
S42-O41	–-	1.585	< N22Cu40O20	–-	72.55			
S42-O43	–-	1.466	< O20Cu40O41	–-	98.19			
S42-O44	–-	1.454	< O41Cu40O19	–-	111.33			
S42-O45	–-	1.587	< Cu40O19C38	–-	112.97			
			< Cu40N22N23	–-	110.75			
			< Cu40N22C15	–-	128.22			
			< Cu40O20C12	–-	102.88			
			< Cu40O20H34	–-	118.16			
			< Cu40O41S42	–-	96.62			
			< O41S42O44	–-	111.43			
			< O43S42O44	–-	116.95			
			< O43S42O45	–-	109.09			
			< O41S42O45	–-	94.76			

In the Cu(II)-PQMHC complex, the copper ion is coordinated by atoms O19, O20, N22, and O41. Upon complexation, the calculated Cu–N and Cu–O bond lengths exhibit elongation, as shown in Table 5. This elongation may be attributed to the relatively low ionic character of the Cu–N and Cu–O bonds within the complex. In addition, the bond lengths near the coordination sites of the PQMHC ligand are generally more elongated compared to the free ligand. Notable elongations include O19-C38 (0.029 Å), O20-C12 (0.001 Å), O20-H34 (0.024 Å), N22-C15 (0.006 Å), and N22-N23 (0.024 Å). Additionally, the N22=C15 bond is longer in the Cu(II)-PQMHC complex than in the uncoordinated ligand, suggesting a reduction in the double bond character upon metal coordination. This interpretation is supported by the observed shift of the N22=C15 absorption band to lower frequencies in the metal complexes, as compared to the free PQMHC ligand (see Table 2). The elongation of bond lengths in the coordination sphere leads to alterations in the corresponding bond angles. The calculated bond angles involving the Cu(II) ion and the coordinating atoms range from 72.55° to 128.22°, indicating a distortion from ideal square planar geometry. Furthermore, the computed dihedral angles around the metal center deviate significantly from 0° and 180°, suggesting that the copper ion does not lie in the same plane as the donor atoms and the rest of the ligand. This confirms that the resulting complex is non-planar in structure.

### Vibrational assignments

**Fig. 8:**
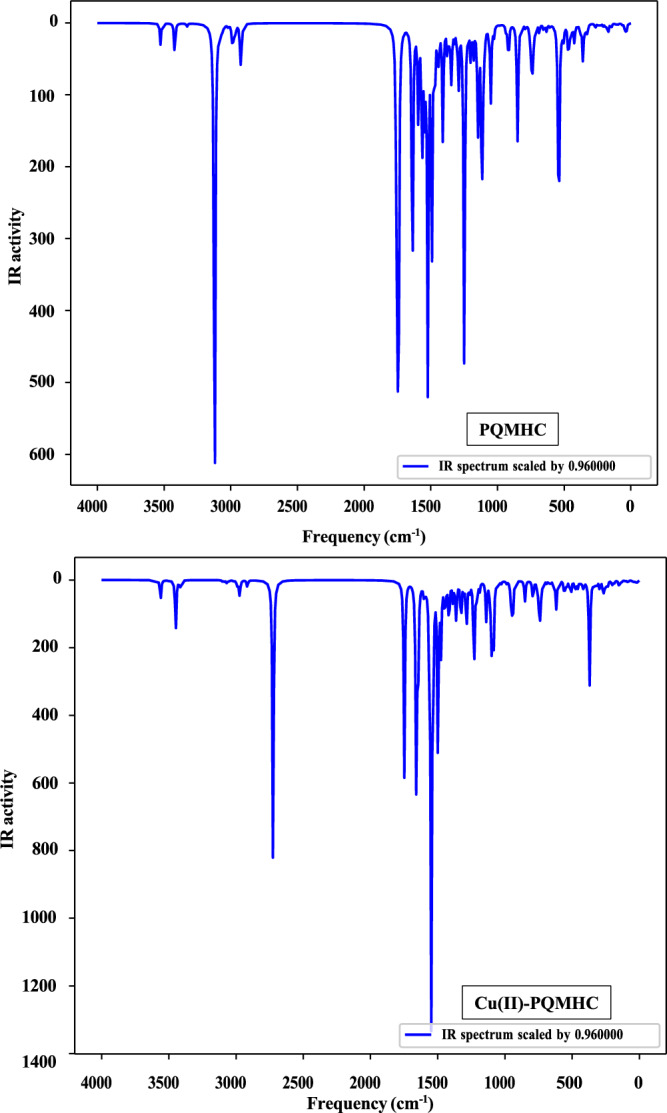
Calculated IR spectra for the PQMHC ligand using B3LYP/6-311G(d,P) and its Copper (II) complex using B3LYP/GENECP.

The PQMHC ligand (C_16_H_14_N_4_O_5_), composed of 39 atoms, exhibits 111 normal vibrational modes and belongs to the C_1_ point group. Similarly, the Cu(II)-PQMHC complex (C_16_H_14_N_4_O_9_CuS), with 45 atoms, displays 129 normal vibrational modes and also conforms to C_1_ symmetry. Vibrational mode assignments were performed based on the FT-IR spectra and correlated with theoretically predicted wavenumbers. To improve agreement with experimental data, the computed vibrational frequencies were scaled using a factor of 0.96^51^, consistent with the level of theory applied. The calculated FT-IR spectra for both the free ligand and its copper complex are presented in Fig. 8.

Experimentally, a broad medium-intensity band was observed at 3440 cm^–1^ (Fig. 2a and b), which was assigned to the O–H stretching vibration. While free O–H stretching typically appears in the 3700 to 3500 cm^-1^ region, hydrogen-bonded O–H stretches are known to shift to the 3550 to 3200 cm^–1^ range^52^. However, neither the optimized molecular structure nor the theoretical analysis supports the existence of an O–H group within the PQMHC ligand or its copper complex. This band likely arises from moisture absorbed from the ambient environment. Additionally, the medium-intensity bands observed between 3400 and 3300 cm^–1^ in the calculated spectrum are attributed to N–H stretching vibrations.

Comparison of computed vibrational frequencies with experimental data shows good overall agreement, validating the chosen basis sets. Minor discrepancies between theoretical and experimental values can be attributed to the fact that computations were carried out for isolated molecules in the gas phase, whereas experimental spectra were obtained under conditions where intermolecular interactions are present^53^.

### Thermodynamical properties

Table 6 details the calculated thermodynamic parameters, including total energy (E_T_), zero-point vibrational energy (ZPVE), entropy (S), nuclear repulsion energy, rotational constants, molar heat capacity at constant volume (C_V_), and thermal energy, all determined at standard conditions (298 K, 1 atm). These calculations were carried out using the B3LYP method with the 6-311G(d, P) basis set for the PQMHC ligand and the GENECP basis set for its copper(II) complex. No scaling or proportional adjustments were applied to the reported values. The Cu(II)-PQMHC complex demonstrated elevated values across all key parameters, indicating enhanced thermodynamic stability compared to the unbound ligand.

**Table 6:** Thermodynamically parameters for the PQMHC ligand computed at the B3LYP/6-311G(d,P), and its Copper (II) complex computed at the B3LYP/GENECP.

Parameters		PQMHC	Cu(II)-PQMHC
Total Energy (E_T_)	(A.U.)	– 1213.61137738	– 2108.94799706
Zero Point Vibrational Energy	(KCal.Mol^-1^)	183.98274	195.66933
Nuclear repulsion energy	(eV)	57,510.693	105,516.388
Rotational constants	(GHz)	0.4712442	0.2344453
		0.1077251	0.0897065
		0.0884948	0.0763612
Entropy (S)	(Cal.Mol^-1^.k^-1^)		
Total		156.905	190.874
Electronic		0.000	1.377
Translational		43.384	44.521
Rotational		35.524	36.546
Vibrational		77.997	108.429
Molar specific heat (C_v_)	(Cal.Mol^-1^.k^-1^ )		
Total		83.004	107.095
Translational		2.981	2.981
Rotational		2.981	2.981
Vibrational		77.042	101.134
Thermal energy (E)	(KCal.Mol^-1^)		
Total		197.565	213.531
Translational		0.889	0.889
Rotational		0.889	0.889
Vibrational		195.788	211.753

To further explore the thermal characteristics of the studied compounds, standard statistical thermodynamic functions, including standard entropy ${S}_{m}^{0}$, heat capacity at constant pressure ${C}_{p, m}^{0}$, and standard enthalpy change $\Delta {H}_{m}^{0}$ were calculated over a temperature range of 100 to 1000 K, based on harmonic vibrational frequencies obtained at the same levels of theory. The data, presented in Table 7, exhibit the expected temperature-dependent increases, attributable to intensified molecular vibrational activity^54^.

**Table 7:** Thermodynamic properties at different temperatures for the PQMHC ligand computed at the B3LYP/6-311G(d,P), and its Copper (II) complex computed at the B3LYP/GENECP.

Parameters	PQMHC			Cu(II)-PQMHC		
T (K)	Sº_m_ (J/mol.K)	Cº_p,m_ (J/mol.K)	ΔHº_m_ (kJ/mol)	Sº_m_ (J/mol.K)	Cº_p,m_ (J/mol.K)	ΔHº_m_ (kJ/mol)
100	400.489	148.632	9.17	462.642	195.684	11.601
150	470.735	201.481	17.922	555.645	267.238	23.19
200	535.957	254.441	29.321	641.879	334.855	38.258
250	598.37	306.72	43.354	723.521	398.636	56.612
298.15	656.601	355.602	59.307	798.728	456.401	77.211
300	658.806	357.439	59.967	801.558	458.545	78.058
350	717.563	405.64	79.056	876.479	514.187	102.395
400	774.698	450.503	100.475	948.519	565.13	129.398
450	830.169	491.56	124.043	1017.788	611.184	158.826
500	883.916	528.703	149.566	1084.36	652.46	190.437
550	935.902	562.096	176.851	1148.307	689.289	223.998
600	986.118	592.05	205.718	1209.718	722.115	259.299
650	1034.588	618.935	236.005	1268.697	751.413	296.151
700	1081.357	643.119	267.566	1325.36	777.636	334.389
750	1126.485	664.942	300.277	1379.828	801.189	373.87
800	1170.04	684.7	334.026	1432.225	822.424	414.469
850	1212.097	702.65	368.717	1482.671	841.639	456.079
900	1252.729	719.007	404.265	1531.279	859.085	498.604
950	1292.01	733.955	440.594	1578.16	874.976	541.961
1000	1330.011	747.651	477.639	1623.415	889.489	586.078

**Fig. 9:**
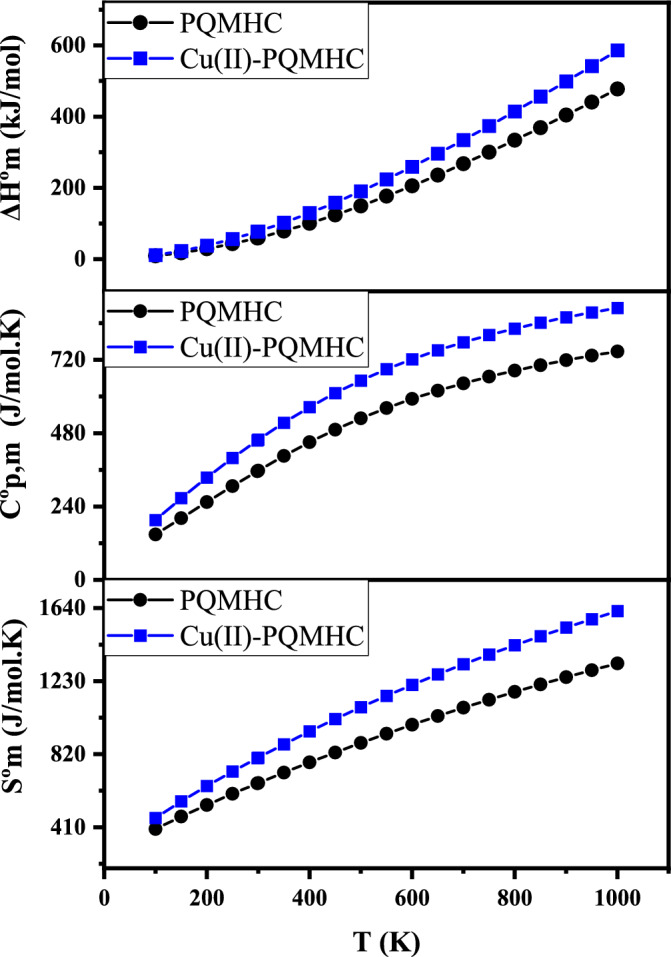
Thermodynamic properties at different temperatures for the studied PQMHC ligand using B3LYP/6-311G(d,P) and its Copper (II) complex using B3LYP/GENECP.

Quadratic polynomial equations were employed to model the relationship between the thermodynamic functions (${S}_{m}^{0}$, ${C}_{p, m}^{0}$, $\Delta {H}_{m}^{0}$) and temperature. The fitted correlation equations achieved high accuracy, with adjusted (R^2^) values exceeding 0.999 for all properties, making them reliable tools for predicting thermodynamic behavior at other temperatures. As illustrated in Fig. 9, the Cu(II)-PQMHC complex consistently exhibits higher thermodynamic values across the entire temperature range compared to the free ligand, underscoring its enhanced thermal stability. These results contribute meaningful insight for future thermodynamic and reactivity studies. The corresponding fitted polynomial equations for the PQMHC ligand are provided below:

1 $${S}_{m}^{0}=267.14637+1.40584-3.44909\times {10}^{-4}{T}^{2} \left({R}^{2}=0.99998\right)$$

2 $${C}_{p, m}^{0}=22.99836+1.28848T-5.69995\times {10}^{-4}{T}^{2} \left({R}^{2}=0.99970\right)$$

3 $$\Delta {H}_{m}^{0}=-17.88045+0.17224T+3.29168\times {10}^{-4}{T}^{2} \left({R}^{2}=0.99947\right)$$

And for the Cu(II)-PQMHC complex:

4 $${S}_{m}^{0}=292.17917+1.84132T-5.15554\times {10}^{-4}{T}^{2} \left({R}^{2}=0.99991\right)$$

5 $${C}_{p, m}^{0}=55.11109+1.55091T-7.28239\times {10}^{-4}{T}^{2} \left({R}^{2}=0.99927\right)$$

6 $$\Delta {H}_{m}^{0}=-25.57024+0.24926T+3.69513\times {10}^{-4}{T}^{2} \left({R}^{2}=0.99945\right)$$

All thermodynamic evaluations were conducted under gas-phase conditions, rendering them unsuitable for solution-phase interpretations. Additional thermodynamic quantities were derived following the second law of thermodynamics, using functional interrelationships and the dynamics of chemical reactions.

### Mulliken atomic charges analyses

**Fig. 10:**
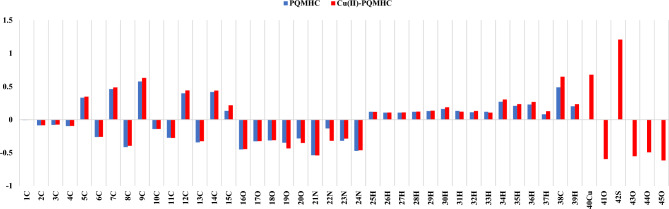
Mulliken atomic charges distributions for the studied PQMHC ligand using B3LYP/6-311G(d,P) and its Copper (II) complex using B3LYP/GENECP.

Mulliken charge analysis provides critical insights into molecular properties such as dipole moment, acidity/basicity, polarizability, and electronic structure. It also elucidates charge redistribution due to atomic displacement and chemical reactivity. As shown in Fig. 10, Mulliken charge distributions were calculated for the PQMHC ligand consuming the B3LYP/6-311G(d, P) basis set and its Cu(II) complex using B3LYP/GENECP. Carbon atoms predominantly exhibit negative charges, except those adjacent to nitrogen and oxygen (e.g., C(5), C(7), C(9), C(12), C(14), C(15), and C(38), which are positively charged due to electron withdrawal. Hydrogen atoms uniformly possess positive charges, while nitrogen and oxygen atoms carry negative charges due to their strong electron-withdrawing nature^55^. Copper and sulfur atoms show positive charges. Notably, O(19), O(20), and N(22) carry more negative charge in the Cu(II) complex than in the free ligand, indicating significant electron redistribution upon complexation.

### Molecular electrostatic potential surface analysis

Molecular electrostatic potential (MEP) analysis visualizes the distribution of electrostatic potential across a molecule’s constant electron density surface in three dimensions. This technique provides a comprehensive view of molecular structure, size, and charge distribution. By mapping regions of varying polarity, MEP highlights areas of electron richness or deficiency using a color scale. Red signifies the most negative potential, marking sites favorable for electrophilic attack, while blue indicates regions of maximum positive potential, associated with nucleophilic interaction. Intermediate shades of orange, yellow, and green represent transitional electrostatic environments^56,57^.

**Fig. 11:**
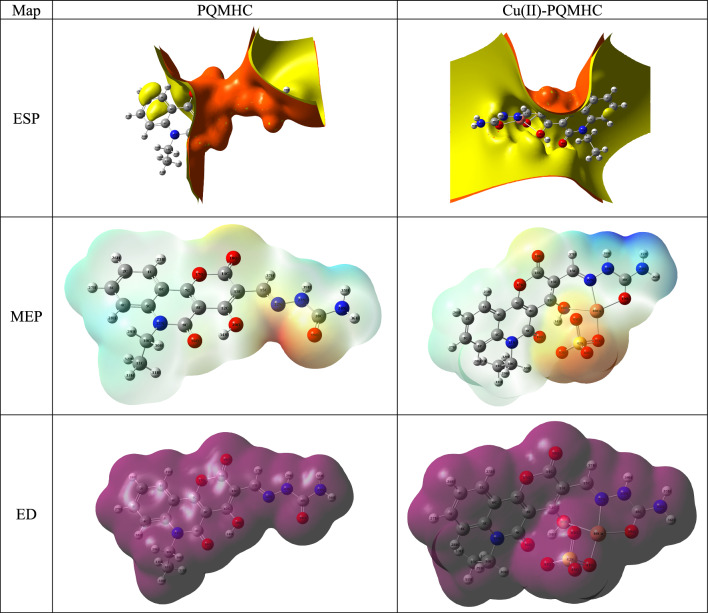
ESP, MEP, and ED maps for the studied PQMHC ligand using B3LYP/6-311G(d,P) and its Copper (II) complex using B3LYP/GENECP.

The electrostatic potential (ESP), molecular electrostatic potential (MEP), and electron density (ED) distributions for the PQMHC ligand and its Cu(II) complex are illustrated in Fig. 11, based on optimized geometries at the same computational level. ESP maps reveal that negative potential regions are localized around nitrogen and oxygen atoms, attributed to lone-pair electron contributions, while positive regions are distributed across the rest of the molecular framework. The ED maps exhibit a generally uniform electron distribution. MEP mapping shows electrophilic attack sites as red regions, localized on nitrogen and oxygen atoms, and nucleophilic sites as blue areas concentrated on hydrogen atoms^58,59^. The blue and red color distributions indicate that each studied compound possesses multiple reactive sites for different types of reactions.

### Natural charges and natural population analysis (NPA)

Table 8 presents detailed data on atomic charge accumulation, electron distribution in the core, valence, and Rydberg subshells, and the natural electronic configurations derived from natural population analysis (NPA) of the PQMHC ligand and its Cu(II) complex. This analysis provides insight into the electronic structure of the active sites by illustrating how electrons are distributed among the atomic orbitals. For the PQMHC ligand, significant negative charges are observed on several electronegative atoms: O16 (– 0.67632), O17 (– 0.51642), O18 (– 0.57830), O19 (– 0.60612), O20 (– 0.60884), N21 (– 0.42185), N22 (– 0.20555), N23 (– 0.44991), and N24 (– 0.83836). From an electrostatic perspective, atoms O19, O20, and N22 exhibit the highest electron density and demonstrate a strong tendency to donate electrons to the central Cu(II) ion in the complex.

**Table 8:** Natural charge, natural population and natural electronic configuration of active sites in the studied PQMHC ligand computed using B3LYP/6-311G(d,P) and Cu-metal in studied Copper (II)-PQMHC complex computed using B3LYP/GENECP.

Compound	Atom No	Natural charge	Natural population				Natural electronic configuration
			Core	Valence	Rydberg	Total	
PQMHC	O16	-0.67632	1.99974	6.66794	0.00864	8.67632	[core]2S(1.70)2p(4.97)
	O17	-0.51642	1.99965	6.50609	0.01068	8.51642	[core]2S(1.60)2p(4.90)
	O18	-0.5783	1.99974	6.5688	0.00976	8.5783	[core]2S(1.69)2p(4.88)3d(0.01)
	O19	-0.60612	1.99975	6.59769	0.00868	8.60612	[core]2S(1.70)2p(4.90)3d(0.01)
	O20	-0.60884	1.99971	6.59993	0.0092	8.60884	[core]2S(1.64)2p(4.96)
	N21	-0.42185	1.99915	5.41062	0.01209	7.42185	[core]2S(1.19)2p(4.22)3p(0.01)
	N22	-0.20555	1.99927	5.18271	0.02357	7.20555	[core]2S(1.37)2p(3.81)3p(0.01)3d(0.01)
	N23	-0.44991	1.99931	5.43347	0.01713	7.44991	[core]2S(1.24)2p(4.19)3p(0.01)
	N24	-0.83836	1.99943	5.82804	0.01088	7.83836	[core]2S(1.38)2p(4.45)3p(0.01)
Cu(II)-PQMHC							
Doublet spin	Cu40	0.94418	17.99744	10.04803	0.01036	28.05582	[core]4S(0.28)3d(9.47)4p(0.30)5p(0.01)
Alpha spin orbitals	Cu40	0.23765	8.99892	5.25857	0.00486	14.26235	[core]4S(0.13)3d(4.98)4p(0.15)
Beta spin orbitals	Cu40	0.70653	8.99852	4.78946	0.00549	13.79347	[core]4S(0.15)3d(4.49)4p(0.15)

Table 9 demonstrates a notable increase in the coordinated atoms’ negative charges following complexation, which can be due to the back-donation of electron density from the central metal ion to the ligand. The table provides detailed values of natural charge redistribution (M → L) for atoms within the coordination sphere, highlighting the electron-accepting nature of electropositive elements such as Cu and S. Moreover, the table quantifies the net electron transfer from ligand to metal (L → M), revealing that the copper center in the complex gains 2.59 electrons, corresponding to a (3d^9^^47^) configuration. These findings, in conjunction with Tables 8 and 9, underscore the strong electronic interaction between the Cu ion and the donor atoms of the ligand.

**Table 9:** Natural charges calculated for the studied Copper (II)-PQMHC complex computed at the B3LYP/GENECP.

Charge transfer	PQMHC	Cu(II)-PQMHC		
		Doublet spin	Alpha spin orbitals	Beta spin orbitals
O16	– 0.67632	– 0.65410	– 0.32704	– 0.32706
Cu40 → O16	–	0.02222	–	–
O17	– 0.51642	– 0.51087	– 0.25542	– 0.25544
Cu40 → O17	–	0.00555	–	–
O18	– 0.5783	– 0.57445	– 0.28729	– 0.28716
Cu40 → O18	–	0.00385	–	–
O19	– 0.60612	– 0.65283	– 0.33746	– 0.31537
Cu40 → O19	–	0.04671	–	–
O20	– 0.60884	– 0.64107	– 0.32053	– 0.32054
Cu40 → O20	–	0.03223	–	–
N21	– 0.42185	– 0.40908	– 0.20459	– 0.20449
Cu40 → N21	–	0.01277	–	–
N22	– 0.20555	– 0.35192	– 0.19430	– 0.15762
Cu40 → N22	–	0.14637	–	–
N23	– 0.44991	– 0.43669	– 0.21845	– 0.21824
Cu40 → N23	–	0.01322	–	–
N24	– 0.83836	– 0.78680	– 0.39375	– 0.39306
Cu40 → N24	–	0.05156	–	–
Cu40	–	0.94418	0.23765	0.70653
PQMHC → Cu40	–	2.59000	–	–
O41	–	– 0.90878	– 0.56170	– 0.34708
S42	–	2.45929	1.23998	1.21931
O43	–	– 0.94947	– 0.48292	– 0.46655
O44	–	– 0.89945	– 0.46140	– 0.43805
O45	–	– 0.92468	– 0.58055	– 0.34414

The results of natural population analysis (NPA) reveal that the PQMHC ligand contains 178 electrons, partitioned into total Lewis (core and valence) and total non-Lewis (valence and Rydberg) contributions. In contrast, the Cu(II) complex accommodates 255 electrons, which are distributed across effective core, core, and valence Lewis’s orbitals, as well as non-Lewis’s components, including valence and Rydberg contributions. Table 10 provides a comprehensive breakdown of these electron distributions and their percentage compositions.

**Table 10:** Natural population of the total electrons in the studied PQMHC ligand computed at the B3LYP/6-311G(d,P), and its Copper (II) complex computed at the B3LYP/GENECP.

Parameters	PQMHC	Cu(II)-PQMHC		
Spin	Singlet	Doublet	Alpha spin orbitals	Beta spin orbitals
Effective Core	–	10.00000	5.00000	5.00000
Core	49.98042 (99.961% of 50)	75.97503 (99.9675% of 76)	37.98773 (99.968% of 38)	37.98729 (99.967% of 38)
Valence Lewis	123.09544 (96.168% of 128)	161.76644 (95.719% of 169)	81.52060 (95.907% of 85)	80.24584 (95.531% of 84)
Total Lewis	173.07586 (97.234% of 178)	247.74147 (97.153% of 255)	124.50833 (97.272% of 128)	123.23314 (97.034% of 127)
Valence non-Lewis	4.53086 (2.545% of 178)	6.57154 (2.5775% of 255)	3.15031 (2.461% of 128)	3.42123 (2.694% of 127)
Rydberg non-Lewis	0.39328 (0.221% of 178)	0.687 (0.2695% of 255)	0.34136 (0.267% of 128)	0.34564 (0.272% of 127)
Total non-Lewis	4.92414 (2.766% of 178)	7.25853 (2.847% of 255)	3.49167 (2.728% of 128)	3.76686 (2.966% of 127)

### Natural bond orbital (NBO) analysis

To examine bonding interactions and electron delocalization within and between molecules, Natural Bond Orbital (NBO) analysis was employed^58^. This approach evaluates charge transfer events from electron-donating orbitals to electron-accepting orbitals. The associated stabilization energies, E^(2)^, which quantify hyper-conjugative effects, were calculated using the second-order perturbative analysis of the Fock matrix elements between donor (i) and acceptor (j) NBOs, according to the following formula:

7 $${E}^{(2)}=\Delta {E}_{ij}={q}_{i}\frac{{F(i, j)}^{2}}{{E}_{j}-{E}_{i}}$$

In this context, ${q}_{i}$ denotes the donor orbital occupancy, ${E}_{i}$ and ${E}_{j}$ are orbital energies (diagonal matrix elements), and $F(i,j)$ corresponds to the off-diagonal NBO Fock matrix element^60^. A higher stabilization energy E^(2)^ signifies stronger donor–acceptor interactions and increased conjugation across the system^61^. This stabilization arises from electron density delocalization from occupied (lone pair or bond) Lewis’s orbitals to unoccupied (Rydberg or anti-bond) non-Lewis’s orbitals. Second-order perturbation theory analysis of the Fock matrix in the NBO basis reveals notable hyper-conjugative interactions, contributing to molecular stability. Table 11 presents key transitions, including bond types, donor and acceptor NBOs, occupancy levels, and E^(2)^ values, demonstrating the stability of both the free ligand and its Cu(II) complex. The significant stabilization observed is attributed to strong interactions between lone pairs and anti-bonding orbitals at opposite ends of the molecule. Based on these findings, the studied compounds can be considered structurally stable.

**Table 11:** Selected Second Order Perturbation Interaction Energy Values Computed in the NBO Basis for the PQMHC ligand computed at the B3LYP/6-311G(d,P), and its Copper (II) complex computed at the B3LYP/GENECP.

PQMHC				Cu(II)-PQMHC					
						Alpha spin orbitals		Beta spin orbitals	
Donor	Acceptor	E^(2)a^(kcal/mol)	Population	Donor	Acceptor	E^(2)a^(kcal/mol)	Population	E^(2)a^(kcal/mol)	Population
π C1–C2	LP(1) C6	44.83	1.68681	π C1–C2	π* C3–C4	11.24	0.84500	11.24	0.84504
π C3–C4	π* C5-N21	34.73	1.70086	π C3–C4	π* C5–N21	17.42	0.84821	17.42	0.84824
π C5–N21	π* C9-O16	32.21	1.80991	π C5-N21	π* C9–O16	16.43	0.90213	16.43	0.90213
π C7–C8	π* C9–O16	28.49	1.70672	π C7–C8	π* C9–O16	13.62	0.84404	13.62	0.84393
π C12–C13	π* C14–O18	32.16	1.69532	π C12–C13	π* C14–O18	16.37	0.83710	16.36	0.83673
LP(1) C6	π* C1–C2	60.25	1.07821	LP(1) C6	π* C1–C2	29.76	0.54204	29.76	0.54196
LP(1) C6	π* C5–N21	252.11	1.07821	LP(1) C6	π* C5–N21	114.48	0.54204	114.47	0.54196
LP(1) C6	π* C7–C8	70.46	1.07821	LP(1) C6	π* C7–C8	36.26	0.54204	36.25	0.54196
LP(1) O16	RY* C9	10.81	1.96718	LP(2) O16	σ* C9–N21	11.35	0.91383	11.34	0.91381
LP(2) O16	σ* C9–N21	23.50	1.84598	LP(2) O16	σ* O20–H34	16.26	0.91383	16.26	0.91381
LP(2) O17	π* C7–C8	40.40	1.73127	LP(2) O17	π* C7–C8	19.36	0.86707	19.35	0.86709
LP(1) O18	RY* C14	16.09	1.97791	LP(2) O17	π* C14–O18	14.53	0.86707	14.53	0.86709
LP(2) O18	σ* C14–O17	41.91	1.81249	LP(2) O18	σ* C14–O17	20.39	0.90714	20.39	0.90707
LP(1) O19	RY* C38	16.89	1.97844	LP(3) O19	LP*(1) C38	114.13	0.80411	115.83	0.80262
LP(2) O19	σ* N23–C38	28.55	1.83636	LP(2) O20	π* C12–C13	24.38	0.88110	24.36	0.88125
LP(2) O19	σ* N24–C38	25.07	1.83636	LP(1) N23	LP*(1) C38	45.99	0.86154	45.95	0.86155
LP(2) O20	π* C12–C13	47.92	1.75982	LP(1) N23	π* C15–N22	10.37	0.86154	10.54	0.86155
LP(1) N23	π* C15–N22	28.87	1.70532	LP(1) N24	LP*(1) C38	50.40	0.88513	50.32	0.88532
LP(1) N23	σ* O19–C38	41.86	1.70532	π* C5–N21	π* C3–C4	38.03	0.36788	38.02	0.36784
LP(1) N24	σ* O19–C38	24.49	1.83708	π* C5–N21	π* C9–O16	26.43	0.36788	26.44	0.36784
π* C5–N21	π* C3–C4	78.25	0.75871	π* C15–N22	π* C12–C13	27.56	0.12151	26.85	0.12122
π* C5–N21	π* C9–O16	62.27	0.75871	LP(2) O19	LP*(6) Cu40	9.71	0.91758	9.93	0.89811
π* C7–C8	π* C12–C13	196.90	0.36080	LP(1) O20	LP*(9,8) Cu40	3.05	0.97499	3.17	0.97486
π* C9–O16	π* C7–C8	118.79	0.40917	LP(1) N22	LP*(6,5) Cu40	18.13	0.92566	19.22	0.89397
π* C12–C13	π* C15–N22	144.68	0.36080	LP(3) O41	LP*(6,5) Cu40	18.38	0.92502	28.40	0.73038
σ* O19–C38	σ* O19–C38	11.68	0.30077						

^a^E^(2)^ means energy of hyper-conjugative interactions (stabilization energy).

LP_(n)_ is a valence lone pair orbital (n) on atom.

### Frontier molecular orbital (FMO)

Frontier molecular orbitals (FMOs), namely the highest occupied molecular orbital (HOMO) and the lowest unoccupied molecular orbital (LUMO), are critical in determining the electronic properties and chemical behavior of molecules. The energy gap between these orbitals provides insight into intramolecular charge transfer (ICT) processes and the interaction between a molecule and its environment^62^. Typically, electronic excitation from the ground state to the first excited state occurs through the transition of an electron from the HOMO, which serves as an electron donor, to the LUMO, functioning as an electron acceptor. The HOMO–LUMO energy gap is a fundamental descriptor of a molecule’s kinetic stability and chemical reactivity^63^. A narrower energy gap facilitates charge transfer and enhances electron mobility between orbitals^64^. Molecules with smaller gaps tend to be more polarizable, exhibit greater chemical reactivity, and possess lower kinetic stability, characteristics commonly associated with chemically “soft” species^65^.

**Fig. 12:**
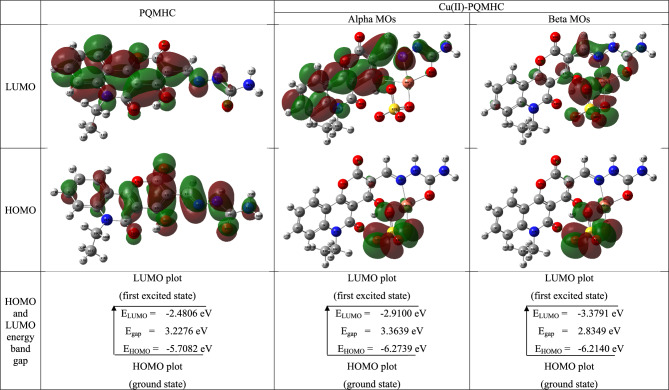
HOMO, LUMO maps, and energy gaps for the studied PQMHC ligand using B3LYP/6-311G(d,P) and its Copper (II) complex using B3LYP/GENECP.

Figure 12 presents three-dimensional (3D) representations of the calculated HOMO and LUMO molecular orbitals for the PQMHC ligand and its Cu(II) complex, obtained at the same theoretical level. The HOMOs are characterized as π-type orbitals, while the LUMOs correspond to π* orbitals. In the visualizations, red and green indicate the positive (regions of maximum electron repulsion) and negative (regions of maximum electron attraction) phases of the orbitals, respectively. Furthermore, the PQMHC ligand possesses a singlet spin multiplicity, as it contains no unpaired electrons. Conversely, the Cu(II) complex exhibits a doublet spin state, resulting from the presence of a singly occupied molecular orbital (SOMO)^66^. The doublet spin state of the Cu(II) complex leads to the splitting of the frontier molecular orbitals into separate alpha and beta orbitals, resulting in two distinct HOMO–LUMO energy gaps.

**Fig. 13:**
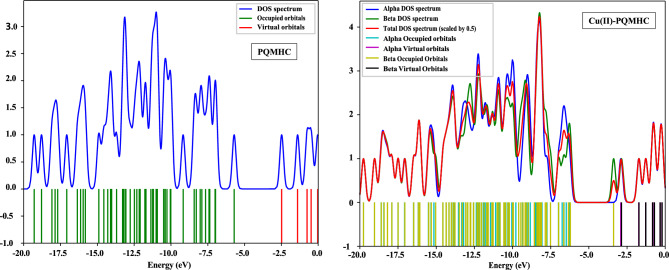
DOS for the studied PQMHC ligand using B3LYP/6-311G(d,P) and its Copper (II) complex using B3LYP/GENECP.

Figure 13 displays the calculated density of states (DOS) spectra for the PQMHC ligand and its Cu(II) complex. The DOS profiles were obtained by applying Gaussian broadening to the discrete molecular orbital energies. These spectra provide insight into the number of electronic states within specific energy intervals for both occupied and unoccupied orbitals.

### Quantum global reactivity descriptors

The global quantum chemical reactivity descriptors for the PQMHC ligand and its Cu(II) complex were computed at the same level of theory. These indices include ionization potential (I), electron affinity (A), global softness (S), chemical hardness (η), electronegativity (χ), chemical potential (μ), and the global electrophilicity index (ω), as defined by the following equations^67,68^:

8 $$I=-{E}_{HOMO}$$

9 $$A=-{E}_{LUMO}$$

10 $$\eta =(I-A)/2$$

11 $$S=1/\eta$$

12 $$V=-\left(I+A\right)/2$$

13 $$\chi =\left(I+A\right)/2$$

14 $$\omega ={V}^{2}/2\eta ={\chi }^{2}/2\eta$$

The calculated quantum chemical global reactivity parameters are summarized in Table 12. The Cu(II)-PQMHC complex exhibits a higher ionization potential than the free PQMHC ligand, indicating a reduced electron-donating ability. Additionally, its lower electron affinity suggests an increased tendency to act as an oxidizing agent. Furthermore, a low chemical hardness value implies an enhanced capability for intramolecular charge transfer, contributing to the complex’s electronic reactivity. The negative values of the chemical potential (μ) for both the PQMHC ligand and its Cu(II) complex suggest that these species are thermodynamically stable and can form spontaneously from their constituent elements, with a low tendency to revert to them. This indicates a high degree of molecular stability and resistance to decomposition. Additionally, the Cu(II)-PQMHC complex exhibits a higher electronegativity compared to the free ligand, reflecting a stronger tendency to attract electrons within the molecular framework. The global electrophilicity index (ω) is also greater for the Cu(II) complex, signifying enhanced electrophilic character and reactivity relative to the uncoordinated ligand^69^.

**Table 12:** Quantum global parameters, spin, energy of HOMO and LUMO, and energy gap of the PQMHC ligand computed at the B3LYP/6-311G(d,P), and its Copper (II) complex computed at the B3LYP/GENECP.

Parameters		PQMHC	Cu(II)-PQMHC	
Spin		Singlet	Doublet	
			Alpha MOs	Beta MOs
Energy of HOMO (E_HOMO_)	(eV)	– 5.7082	– 6.2739	– 6.2140
Energy of LUMO (E_LUMO_)	(eV)	– 2.4806	– 2.9100	– 3.3791
Energy gap (E_gap_)	(eV)	3.2276	3.3639	2.8349
Ionization potential (I)	(eV)	5.7082	6.2739	6.2140
Electron affinity (A)	(eV)	2.4806	2.9100	3.3791
Chemical hardness (η)	(eV)	1.6138	1.6820	1.4175
Global softness (S)	(eV^-1^)	0.6197	0.5945	0.7055
Chemical potential (V)	(eV)	– 4.0944	– 4.5920	– 4.7966
Electronegativity (χ)	(eV)	4.0944	4.5920	4.7966
Global electrophilicity index (ω)	(eV)	5.1940	6.2683	8.1155

### Non-quantum global reactivity descriptors

The non-quantum global reactivity descriptors for the PQMHC ligand and its Cu(II) complex were calculated using the same theoretical framework. These descriptors include surface area grid (SAG), molar volume (MV), octanol/water partition coefficient (log P), molar refractivity (MR), hydration energy (HE), polarizability (Pol), and molecular weight (MW), as summarized in Table 13. Molecular polarizability (Pol) refers to the ability of a molecule’s electronic cloud to deform in response to an external electric field, such as that from incident light. It is largely influenced by molecular volume, which in turn affects the transport properties of molecules. Molar refractivity (MR), a steric parameter, is influenced by the three-dimensional arrangement of aromatic rings and is directly related to the molecular volume^70^.

**Table 13:** Non-quantum global parameters of the PQMHC ligand computed at the B3LYP/6-311G(d,P), and its Copper (II) complex computed at the B3LYP/GENECP.

Parameters	PQMHC	Cu(II)-PQMHC
SAG (Å^2^)	535.55	590.01
MV(Å^3^)	894.25	1025.24
Log P (Octane/H_2_O)	6.34	8.76
MR (Å^3^)	35.00	50.06
HE (KCal/Mol)	– 21.31	– 29.31
Pol (Å^3^)	33.37	35.83
MW (amu)	342.31	501.91

As shown in Table 13, polarizability, molar refractivity, and surface area values generally increase with molecular size and weight. Furthermore, the data indicate that higher hydrophobicity, as reflected by elevated log P values, correlates with reduced hydration energy. Variations in hydration energy are attributed to changes in the number and strength of hydrogen bonding interactions between donor and acceptor groups^70^. Notably, the Cu(II)-PQMHC complex exhibits higher values across all non-quantum descriptors compared to the free ligand, suggesting an overall increase in molecular size, polarizability, and hydrophobicity upon coordination.

### Non-linear optical (NLO) analysis

To explore the relationship between molecular structure and non-linear optical (NLO) properties, the polarizabilities and first-order hyperpolarizabilities of the PQMHC ligand and its Cu(II) complex were computed using a consistent theoretical framework employing the finite field approach. Dipole moment, polarizability, and hyperpolarizability components were extracted from frequency job output files generated by Gaussian 09W and are summarized in Table 14. The total dipole moment (µ_tot_), mean polarizability (α_tot_), polarizability anisotropy (Δα), and total first-order hyperpolarizability (β_tot_) were calculated using established expressions^71,72^:

**Table 14:** Total static dipole moment (μ), the mean polarizability (˂α˃), the anisotropy of the polarizability (Δα), the mean first-order hyperpolarizability (˂β˃), the depolarization ratio (DR), and the Hyper-Rayleigh scattering (β_HRS_) of the PQMHC ligand computed at the B3LYP/6-311G(d,P), and its Copper (II) complex computed at the B3LYP/GENECP.

Components	Urea	PQMHC	Cu(II)-PQMHC
μ_x_ (D)		5.8152	– 2.0344
μ_y_ (D)		– 1.3629	– 8.5081
μ_z_ (D)		1.0838	– 6.7770
μ (Debye)	1.3197	6.0703	11.0659
α_XX_ (a.u.)		– 94.8332	– 84.6298
α_XY_ (a.u.)		20.9184	33.7820
α_YY_ (a.u.)		– 153.6112	– 208.7903
α_ZZ_ (a.u.)		– 144.5683	– 206.0816
α_YZ_ (a.u.)		– 0.1035	– 8.5906
α_XZ_ (a.u.)		– 7.4859	14.0889
˂α > (a.u.)		– 131.0042	– 166.5006
˂α > × 10^−24^ (esu)		– 19.4148	– 24.6754
Δα (a.u.)		66.9775	139.0231
Δα × 10^−24^ (esu)	3.8312	9.9261	20.6032
β_xxx_ (a.u.)		– 154.8519	– 431.8774
β_xxy_ (a.u.)		– 95.8470	– 112.6393
β_xyy_ (a.u.)		58.1398	– 46.7562
β_yyy_ (a.u.)		35.9990	39.6958
β_xxz_ (a.u.)		62.2185	– 14.8136
β_xyz_ (a.u.)		4.3333	– 27.4015
β_yyz_ (a.u.)		2.3929	– 52.2211
β_xzz_ (a.u.)		– 25.4373	– 31.2372
β_yzz_ (a.u.)		8.0197	– 24.1772
β_zzz_ (a.u.)		1.2744	– 27.4439
˂β˃ (a.u.)		148.1472	527.5669
˂β˃ × 10^−30^ (esu)	0.1947	1.2799	4.5578
DR		4.1954 × 10^−4^	3.4322
β_HRS_		62.2316	31.1867

15 $${\mu }_{tot}=\sqrt{{\mu }_{x}^{2}+{\mu }_{y}^{2}+{\mu }_{z}^{2}}$$

16 $${\alpha }_{tot}=\frac{{\alpha }_{xx}+{\alpha }_{yy}+{\alpha }_{zz}}{3}$$

17 $$\Delta \alpha =\sqrt{\frac{{\left({\alpha }_{xx}-{\alpha }_{yy}\right)}^{2}+{\left({\alpha }_{yy}-{\alpha }_{zz}\right)}^{2}+{\left({\alpha }_{zz}-{\alpha }_{xx}\right)}^{2}+6{\alpha }_{xy}^{2}+6{\alpha }_{yz}^{2}+6{\alpha }_{xz}^{2}}{2}}$$

18 $${\beta }_{tot}=\sqrt{{\left({\beta }_{xxx}+{\beta }_{xyy}+{\beta }_{xzz}\right)}^{2}+{\left({\beta }_{yyy}+{\beta }_{yzz}+{\beta }_{yxx}\right)}^{2}+{\left({\beta }_{zzz}+{\beta }_{zxx}+{\beta }_{zyy}\right)}^{2}}$$

Elevated values of dipole moment, molecular polarizability, and hyperpolarizability are recognized as key contributors to enhanced non-linear optical (NLO) activity. Within the scope of second-order NLO properties, emphasis was placed on hyper-Rayleigh scattering (β_HRS_) and the depolarization ratio (DR)^73^, both evaluated using the following equations:

19 $${\beta }_{HRS}=\sqrt{{\beta }_{zzz}^{2}+{\beta }_{zxx}^{2}}$$

20 $$DR=\frac{{\beta }_{zzz}^{2}}{{\beta }_{zxx}^{2}}$$

The variations in β_HRS_ and DR values can be attributed to structural factors; specifically, the low β_HRS_ and high DR values indicate an extended bond length between the metal center in the complex and the donor atoms of the ligand, supporting the occurrence of physical adsorption, strong complexation, and high catalytic efficiency^74^. The computed values of (µ_tot_), (α_tot_), (Δα), (β_tot_), (β_HRS_), and (DR) for the PQMHC ligand and its Cu(II) complex are summarized in Table 14. These values were converted into electrostatic units (esu) using the following conversion factors: α (1 a.u. = 0.1482 × 10^−24^ esu) and β (1 a.u. = 8.6393 × 10^−33^ esu). Urea was selected as a reference compound due to the absence of experimental NLO data for the studied ligand and complex. It is commonly employed as a benchmark in such comparisons, with reported values of (µ = 1.3197 D, Δα = 3.8312 × 10^−24^ esu, and β = 0.1947 × 10^−33^ esu)^75,76^.

For the PQMHC ligand, the computed total static dipole moment (µ_tot_) is 6.0703 Debye, approximately 4.6 times that of urea (1.3732 D), which is often used as a benchmark NLO reference material. The dominant component lies along the x-axis (5.8152 D). Upon complexation with Cu(II), µ_tot_ increases substantially to 11.0659 Debye **(~ **8.4 times that of urea), with the largest component along the y-axis (– 8.5081 D). This pronounced enhancement in dipole moment is attributed to the strong electron-withdrawing effect of the nitro group (–NO₂), which promotes intramolecular charge transfer and increases overall molecular polarity. Such polarity is a well-recognized factor in improving NLO performance and optoelectronic activity ^77,78^.

The calculated anisotropy of polarizability (Δα) for the PQMHC ligand is 9.9261 × 10⁻^24^ esu, approximately 2.6 times higher than urea. For the Cu(II)-PQMHC complex, Δα increases to 20.6032 × 10⁻^24^ esu, equivalent to 5.4 times the urea value, indicating a substantial rise in the degree of electronic cloud distortion upon complexation.

The mean first-order hyperpolarizability (β_tot_) of the PQMHC ligand is 1.2799 × 10⁻^3^⁰ esu, about 6.6 times greater than urea (0.1947 × 10⁻3⁰ esu). Remarkably, the Cu(II)-PQMHC complex displays a β_tot_ value of 4.5578 × 10⁻^3^⁰ esu, corresponding to 23.4 times the urea standard. When compared with literature-reported β values for structurally related Cu(II) semicarbazone complexes, typically in the range of 1.5–3.0 × 10⁻^3^⁰ esu, the present Cu(II)-PQMHC system demonstrates a clear advantage, surpassing the upper range by more than 50%. Furthermore, these β values approach or exceed those of some reported organic push–pull NLO chromophores, placing the complex among the higher-performing candidates in its class.

Additionally, the low depolarization ratio (DR) and high hyper-Rayleigh scattering (β_HRS_) values for both the ligand and complex indicate a shorter metal–ligand bond length, implying strong coordination, high selectivity, and enhanced catalytic activity^73,79^. The reduced total hydrogen-bond contribution further confirms selective Cu(II) complex formation with minimal free ligand interference. Overall, these findings establish the Cu(II)-PQMHC complex as a superior NLO-active material, with properties significantly exceeding those of typical semicarbazone-based Cu(II) complexes and aligning with or surpassing well-known benchmark chromophores.

### Electronic absorption spectra

**Fig. 14:**
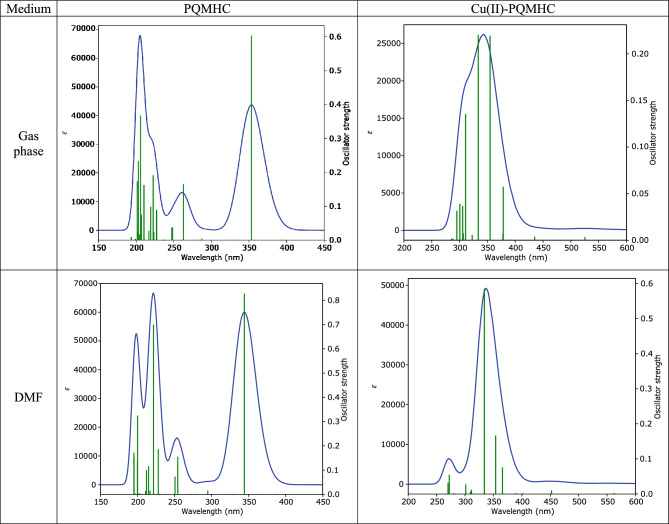
Calculated UV–Vis spectra for the studied PQMHC ligand using CAM-B3LYP/6-311G(d,P) and its Copper (II) complex using CAM-B3LYP/GENECP in gas phase and DMF.

Time-dependent DFT calculations (CAM-B3LYP/6-311G(d, P) for the PQMHC ligand and CAM-B3LYP/GENECP for the Cu(II)–PQMHC complex) were performed to elucidate the nature of the electronic transitions. Figure 14 displays the simulated absorption spectra in both gas phase and DMF, while Tables 15 and 16 summarize the vertical excitation energies, oscillator strengths, and orbital contributions.

**Table 15:** Computed excitation energies (in eV), electronic transition configurations, and oscillator strengths ^a^ (ƒ) for the optical transitions of the absorption bands in the UV–vis regions (involving HOMOs) of the PQMHC ligand at the CAM-B3LYP /6-311G(d,P).

Medium	Transition states	E (eV)	Transition types	λ_max_ (nm)	(ƒ)	Configuration composition corresponding transition orbital
Gas phase	S0—> S4	3.5102	n–π*	353.21	0.6031	– 0.10(86—> 90); 0.67(89—> 90)
	S0—> S11	4.7170	π–π*	262.84	0.1653	0.12(83—> 90); – 0.14(86—> 90); – 0.22(88—> 90); – 0.13(89—> 90); 0.56(89—> 91); 0.25(89—> 92)
	S0—> S22	5.5724	π–π*	222.50	0.1916	0.17(82- > 90); 0.21(83—>90); 0.22(86—>90); 0.11(87—>90); – 0.13(88—>91); 0.10(89—>91); 0.40(89—>93); – 0.30(89—>94)
	S0—> S32	5.8899	π–π*	210.50	0.1632	– 0.12(79—>90); 0.17(80—>90); 0.21(83—>90); – 0.13(86—>91); .12(86—>92); 0.37(88—>91); 0.22(88—>93); – 0.15(89—>93); – 0.33(89—>94)
	S0—> S34	6.0205	π–π*	205.94	0.3681	– 0.12(80—>91); 0.17(82—>90); 0.11(82—>94); 0.13(83—>90); – 0.14(83—>91); 0.14(85—>91); – 0.15(86—>90); 0.36(88—>91); – 0.13(88—>93); – 0.11(89—>92); 0.26(89—>93); 0.23(89—>94)
	S0—> S36	6.1104	π–π*	202.91	0.2338	0.19075(80—>90); – 0.20359(81—>90); – 0.24942(83—>90); – 0.16159(83—>91); – 0.1021(84—>90); 0.36504(86—>91); 0.17736(88—>92); 0.13063(89—>91); – 0.15722(89—>94); 0.18102(89—>96)
	S0—> S38	6.1565	π–π*	201.39	0.1743	– 0.32(80—>90); 0.36(81—>90); 0.25(86—>91); 0.11(86—>92); 0.12(88—>91); – 0.18(89—>92); – 0.23(89—>93)
DMF	S0—> S4	3.6012	n–π*	344.29	0.8279	– 0.12(87—>90); – 0.11(88—>90); 0.67(89—>90)
	S0—> S12	4.8748	π–π*	254.34	0.1553	– 0.13(81—>90); 0.16(87—>90); 0.17(88—>90); 0.14(89—>90); 0.59(89—>91); – 0.17(89—>92)
	S0—> S18	5.4380	π–π*	227.99	0.1866	– 0.13(81—>90); – 0.20(84—>90); 0.18(88—>91); – 0.10(89—>91); – 0.27(89—>92); 0.47(89—>93); 0.23(89—>94)
	S0—> S21	5.5969	π–π*	221.52	0.6993	0.30(87—>90); 0.48(88—>91); 0.16(88—>92); – 0.13(89—>91); – 0.25(89—>93)
	S0—> S24	5.7706	π–π*	214.85	0.1165	0.14(81—>90); – 0.10(82—>90); 0.53(84—>90); 0.27(87—>91); 0.20(88—>91); – 0.16(89—>92)
	S0—> S35	6.1973	π–π*	200.06	0.3246	0.21(81—>90); – 0.22(81—>91); – 0.24(84—>90); 0.30(87—>91); – 0.11(87—>92); – 0.22(88—>92); 0.12(88—>93); – 0.18(88—>94); – 0.13(89—>93); 0.27(89—>94)
	S0—> S36	6.2050	π–π*	199.81	0.1392	0.56(81—>90); – 0.11(84—>90); 0.15(84—>91); 0.10(88—>92); 0.11(88—>94); 0.17(89—>93); – 0.14(89—>94)
	S0—> S38	6.3475	π–π*	195.33	0.1546	– 0.16(81—>91); 0.27(87—>91); – 0.16(87—>92); 0.11(87—>93); – 0.12(87—>94); 0.49(88—>92); 0.19(88—>94); 0.14(89—>92)
	S0—> S39	6.3511	π–π*	195.22	0.1717	– 0.14(79—>90); 0.13(81—>91); – 0.15(87—>91); 0.11(87—>93); – 0.13(87—>94); – 0.13(88—>92); – 0.25(88—>93); 0.26(88—>94); 0.16(89—>92); – 0.15(89—>93); 0.43(89—>94)

**Table 16:** Computed excitation energies (in eV), electronic transition configurations, and oscillator strengths ^a^ (ƒ) for the optical transitions of the absorption bands in the UV–vis regions (involving HOMOs) of the Copper (II)-PQMHC complex at the CAM-B3LYP/GENECP.

Medium	Transition states	E (eV)	Transition types	λ_max_ (nm)	(ƒ)	Configuration composition corresponding transition orbital
Gas phase	S0—> S9	3.2754	n–π*	378.53	0.0574	0.22(120A—> 124A); 0.19(123A—> 124A); – 0.11(104B—> 123B); 0.15(105B—> 123B); – 0.12(113B—> 123B); – 0.11(115B—> 123B); – 0.40(116B—> 123B); – 0.17(116B—> 124B); 0.31(117B—> 123B); 0.10(117B—> 124B); 0.50(118B—> 123B); 0.23(118B—> 124B); – 0.11(119B—> 124B); – 0.12(119B—> 125B); 0.11(120B—> 123B); – 0.22(121B—> 123B); 0.17(121B—> 124B); – 0.17(122B—> 124B)
	S0—> S11	3.4937	n–π*	354.88	0.2195	0.21(122A—> 124A); 0.37(123A—> 124A); 0.15(102B—> 123B); 0.13(113B—> 123B); 0.31(116B—> 123B); 0.12(116B—> 124B); 0.10(117B—> 124B); – 0.19(118B—> 123B); – 0.12(118B—> 124B); – 0.20(119B—> 123B); 0.37(120B—> 123B); – 0.35(121B—> 123B); 0.43(122B—> 123B)
	S0—> S12	3.7151	n–π*	333.73	0.2204	– 0.22(122A—> 124A); – 0.31(123A—> 124A); 0.10(109B—> 123B); – 0.25(116B—> 123B); – 0.12(116B—> 124B); 0.13(117B—> 123B); – 0.15(119B—> 123B); – 0.10(119B—> 124B); 0.22(120B—> 123B); 0.28(120B—> 124B); – 0.13(121B—> 123B); – 0.42(121B—> 124B); 0.17(122B—> 123B); 0.48(122B—> 124B)
	S0—> S14	3.9883	n–π*	310.87	0.1356	0.31(120A—> 124A); – 0.10(121A—> 124A); – 0.19(122A—> 124A); – 0.11(113B—> 123B); 0.56(116B—> 123B); 0.24(116B—> 124B); 0.35(117B—> 123B); 0.22(118B—> 123B); 0.13(118B—> 124B); – 0.18(119B—> 123B); 0.22(119B—> 124B); – 0.14(120B—> 123B); 0.13(120B—> 124B); – 0.13(121B—> 124B)
	S0—> S17	4.1258	π–π*	300.51	0.0389	– 0.25(120A—> 124A); 0.18(121A—> 124A); 0.25(122A—> 124A); – 0.27(123A—> 124A); 0.12(116B—> 123B); 0.18(119B—> 123B); – 0.34(119B—> 124B); – 0.21(120B—> 123B); 0.50(120B—> 124B); – 0.15(120B—> 125B); 0.19(122B—> 123B); – 0.37(122B—> 124B)
DMF	S0—> S10	3.3886	n–π*	365.88	0.0755	– 0.30(123A—> 124A); – 0.24(96B—> 123B); 0.10(98B—> 123B); 0.29(115B—> 123B); 0.28(116B—> 123B); 0.33(117B—> 123B); – 0.30(118B—> 123B); 0.53(119B—> 123B); – 0.29(122B—> 124B)
	S0—> S11	3.5032	n–π*	353.92	0.1669	0.39(123A—> 124A); 0.21(94B—> 123B); 0.12(95B—> 123B); – 0.31(96B—> 123B); – 0.19(109B—> 123B); 0.10(110B—> 123B); 0.12(114B—> 123B); – 0.12(115B—> 123B); – 0.17(116B—> 123B); – 0.18(117B—> 123B); – 0.32(118B—> 123B); 0.36(119B—> 123B); – 0.21(120B—> 123B); – 0.11(121B—> 123B); 0.41(122B—> 124B)
	S0—> S12	3.7126	n–π*	333.95	0.5854	0.46(123A—> 124A); 0.16(109B—> 123B); – 0.21(110B—> 123B); 0.11(113B—> 123B); – 0.13(114B—> 123B); 0.31(115B—> 123B); 0.37(116B—> 123B); 0.33(117B—> 123B); 0.47(122B—> 124B)
	S0—> S19	4.5546	π–π*	272.22	0.0547	– 0.11(117A—> 124A); – 0.11(121A—> 126A); – 0.13(122A—> 125A); 0.15(122A—> 127A); – 0.20(123A—> 125A); 0.51(110B—> 123B); – 0.17(111B—> 123B); – 0.29(112B—> 123B); – 0.28(114B—> 123B); 0.23(117B—> 123B); – 0.17(120B—> 123B); – 0.27(120B—> 124B); 0.11(121B—> 123B); – 0.12(121B—> 127B); 0.12(122B—> 125B); – 0.16(122B—> 126B)

*A* Alpha spin orbital, *B* Beta spin orbital.

In the gas phase, the intense absorption band at 353.21 nm (91% HOMO → LUMO) corresponds to a π → π* intra-ligand (IL) transition within the conjugated heteroaromatic framework. The band at 262.84 nm involves HOMO → LUMO + 1 (62%), HOMO–1 → LUMO (10%), and HOMO → LUMO + 2 (13%), indicative of mixed π → π* and n → π* character from the azomethine nitrogen lone pairs to the ligand π* system. Shorter-wavelength features, such as the 222.50 nm (HOMO → LUMO + 3, HOMO → LUMO + 4) and 210.50 nm (HOMO–1 → LUMO + 1, HOMO → LUMO + 4) bands, reflect higher-energy π → π* excitations with partial charge redistribution along the nitro-substituted furan moiety, enhancing molecular polarization. The transitions at 205.94**,** 202.91**,** and 201.39 nm involve deeper-lying HOMOs (e.g., HOMO–9, HOMO–8) and correspond to localized π → π* excitations within aromatic segments, contributing to the broad UV absorption profile.

In DMF, solvent stabilization slightly red shifts the main π → π* IL band to 344.29 nm (HOMO → LUMO, 89%). The 254.34 nm band (HOMO → LUMO + 1, 70%) retains π → π* character, while 227.99 nm and 221.52 nm involve HOMO → LUMO + 3 and HOMO–1 → LUMO + 1 excitations, mixing π → π* and n → π* transitions. The 214.85 nm and 200.06 nm bands incorporate contributions from oxygen/nitrogen lone pairs to ligand π* orbitals, supporting solvent-induced modulation of IL transitions—relevant for tuning absorption in device environments.

For the complex, the presence of Cu(II) (d⁹0.4 configuration) introduces metal-centered (MC) and charge-transfer states in both α-spin (A) and β-spin (B) manifolds.

In the gas phase, the 378.53 nm band arises from HOMO–6(B) → LUMO(B) (16%) and HOMO–4(B) → LUMO(B) (25%), characteristic of LMCT from ligand π-orbitals into Cu(II)-centered acceptor orbitals. The 354.88 nm band combines IL π → π^*^ contributions (HOMO(A) → LUMO(A), 14%) with LMCT components from HOMO(B) and HOMO–1(B) into metal-based LUMOs. The 333.73 nm and 310.87 nm absorptions exhibit mixed IL and LMCT nature, while the 300.51 nm band (HOMO–2(B) → LUMO + 1(B), 25%) shows strong ligand-to-metal donation, highlighting efficient charge transfer pathways.

In DMF, the main LMCT band appears at 365.88 nm (HOMO–3(B) → LUMO(B), 29%), red-shifted relative to the gas phase, suggesting solvent-assisted stabilization of CT states. The 353.92 nm and 333.95 nm bands continue to display mixed IL/LMCT character, with notable HOMO(B) → LUMO + 1(B) contributions enhancing delocalization between the ligand and Cu(II) center. The 272.22 nm feature (HOMO–12(B) → LUMO(B), 26%) is primarily LMCT, reinforcing the role of deeper ligand orbitals in feeding electron density to the metal.

The PQMHC ligand predominantly shows π → π* IL transitions, while complexation with Cu(II) introduces pronounced LMCT states in the visible region, enhancing absorption breadth and intensity. Such LMCT bands are advantageous for optoelectronic applications, as they promote strong intramolecular charge transfer and broaden spectral coverage into the near-visible region—beneficial for NLO devices, light-harvesting systems, and photonic switches. The combined IL and LMCT character also facilitates directional charge separation, a desirable feature for molecular electronic and photovoltaic platforms.

## Conclusion

A novel Cu(II)-PQMHC complex was synthesized and characterized using elemental analysis, infrared (IR), mass spectrometry (MS), UV–Vis spectroscopy, electron spin resonance (ESR), molar conductance, and thermal analysis. Spectroscopic data indicated that the ligand behaves as a tridentate (O_2_N) donor, coordinating through the keto oxygen, azomethine nitrogen, and hydroxyl oxygen atoms, forming a square planar geometry around the metal center.

Density Functional Theory (DFT) calculations were employed to optimize the molecular structures at the B3LYP/6-311G(d, P) level for the ligand and at the B3LYP/GENECP level for the Cu(II) complex. The theoretical data aligned well with the experimental findings. The optimized geometry of the Cu(II)-PQMHC complex was found to be non-planar, as evidenced by the dihedral angles. Additional computational analyses, including molecular electrostatic potential (MEP), thermodynamic parameters, non-linear optical (NLO) properties, total dipole moment, frontier molecular orbitals (HOMO–LUMO), natural bond orbital (NBO) analysis, natural population analysis (NPA), and global/local reactivity descriptors, were also performed. Time-dependent DFT (TD-DFT) calculations at the CAM-B3LYP level were used to investigate the electronic absorption spectra of the free ligand and its Cu(II) complex.

## Supplementary Information

Supplementary Information.

## Data Availability

All data generated or analysed during this study are included in this published article [and its supplementary information files].
