# Supplementary material for: Exploring a novel copper(II) semicarbazone–pyranoquinoline complex: synthesis, spectroscopic profiling, and DFT insights
Source: Sci Rep. 2025 Nov 18;15:40355. doi: 10.1038/s41598-025-26205-8 (PMC12627528; doi:10.1038/s41598-025-26205-8)
Supplement: Supplementary file 1 — Supplementary Information. [file 41598_2025_26205_MOESM1_ESM.docx]

**Exploring a Novel Copper(II) Semicarbazone-Pyranoquinoline Complex: Synthesis, Spectroscopic Profiling, and DFT Insights**

A.A. El-Saady ^a,^ *, Magdy A. Ibrahim ^b^, M.M. El-Nahass ^a^, Omima M.I. Adly ^b^, A.A.M. Farag ^a^, Nesma Salah ^b^

^a^ Thin Film Laboratory, Physics Department, Faculty of Education, Ain Shams University, Roxy, Cairo 11757, Egypt

^b^ Department of Chemistry, Faculty of Education, Ain Shams University, Roxy, Cairo 11757, Egypt

**Table S1.** Calculated vs. experimental weight loss data for the thermal decomposition of the Cu(II)-PQMHC complex.

| Decomposition Step | Temperature Range (°C) | Peak Temp. (°C) | Assigned Fragment Loss | Calculated Weight Loss (%) | Experimental Weight Loss (%) |
| --- | --- | --- | --- | --- | --- |
| Step 1 | 30–148 | 60 | –0.5 EtOH (lattice) | 4.38 | 4.27 |
| Step 2 | 148–300 | 264 | –H₂SO₄ | 18.70 | 18.98 |
| Step 3 | 300–1000 | 445 | –C₁₂H₉N₄O₄ | 52.09 | 51.58 |
| Residue | — | — | CuO + C₄H₄ | 25.10 | 24.62 |
